# Regulation on microbial composition,serotonergic synapse, and apoptotic signaling pathway by extracts from*Sonchus brachyotus* DC. (SBE) to improve ethanol-induced acute oxidative stress in mice

**DOI:** 10.1186/s40168-025-02221-8

**Published:** 2025-10-29

**Authors:** Yang Juan, Tian Wei, Zhou Weiwei, Shi dongdong, Zhang Mengjie, Wang Xuefeng, Li Xianglong, Sun Yuelong, Zhao Jinhua, Li Xiumei

**Affiliations:** 1 Key Laboratory of Feed Biotechnology, Ministry of Agriculture and Rural Affairs, Institute of Feed Research of CAAS, Beijing, 100081 China; 2 Tobacco Research Institute of Chinese Academy of Agricultural Sciences, Qingdao, Shandong 266101 China; 3 State Key Laboratory of Food Nutrition and Safety, Key Laboratory of Industrial Fermentation Microbiology of Ministry of Education & Tianjin Key Laboratory of Industrial Microbiology, College of Biotechnology, Tianjin University of Science and Technology, Tianjin, 300457 China; 4 Hailu Microecological (Beijing) Biotechnology Co., Ltd, Beijing, 100081 China

**Keywords:** *Sonchus brachyotus* DC., Oxidative stress, Bacteria, Fungi, Serotonergic synapse pathway, Apoptosis signaling pathway

## Abstract

**Background:**

Oxidative stress has been firmly established as a pivotal contributor to the pathogenesis of inflammatory bowel disease, diabetes mellitus, Alzheimer’s disease, and other multifactorial disorders. Our previous findings have demonstrated the extracts from *Sonchus brachyotus* DC. (SBE) mitigates intestinal oxidative stress through interactions between the oxidative stress biomarkers and gut microbiota. However, we did not focus on the mechanism by which SBE exerts antioxidant stress effects through regulating metabolites and genes, nor the correlation between the two and gut microbiota. Therefore, this study aimed to elucidate the underlying mechanism by which SBE mitigates oxidative stress through the gut microbiota, metabolites, and genes.

**Results:**

Supplementation with SBE exerts a promising regulatory effect on oxidative stress by modulating key oxidative stress biomarkers (e.g., GSH, SOD, etc.) in serum, intestine, liver, and brain tissues in ethanol-model mice. And the SBE treatment exhibited a notable reparative effect on intestine, liver, and brain tissue damage. Concomitantly, 16S rRNA and ITS sequencing revealed significant alterations in the composition of intestinal bacteria and fungi in SBE-treated mice, suggesting the restoration of gut microbiota homeostasis. Spearman correlation analysis further indicated significant associations (*p* < 0.05) between gut microbes, particularly fungal genera, and oxidative stress biomarkers. Notably, the abundance of specific fungal genera (*Alternaria* and *Pichia*), the levels of 14,15-DiHETrE, 5-Hydroxyindole-3-acetic acid, and prostaglandin C2 key metabolites of the serotonergic synapse pathway, and the expression of *Fas* and *Tnfsf10* key genes of apoptosis signaling pathway were significantly correlated (*p* < 0.05) based on the constructed correlation network. This mechanism likely triggers coordinated changes in metabolites and gene expression associated with the serotonergic synapse and apoptosis signaling pathways, ultimately leading to multi-targeted amelioration of oxidative stress. Molecular docking further revealed that trigonelline, mesaconic acid, and salicylic acid, bioactive components of SBE, may exhibit considerable binding affinity with *Fas* and *Tnfsf10*, providing a potential structural basis for SBE’s regulatory effects on oxidative stress via modulation of the apoptotic signaling.

**Conclusions:**

The antioxidant effects of SBE likely involve multi-pathway and multi-target mechanisms, consistent with the combinatorial properties of its herbs constituents. These findings lays a foundation for subsequent research.

Video Abstract

**Graphical Abstract:**

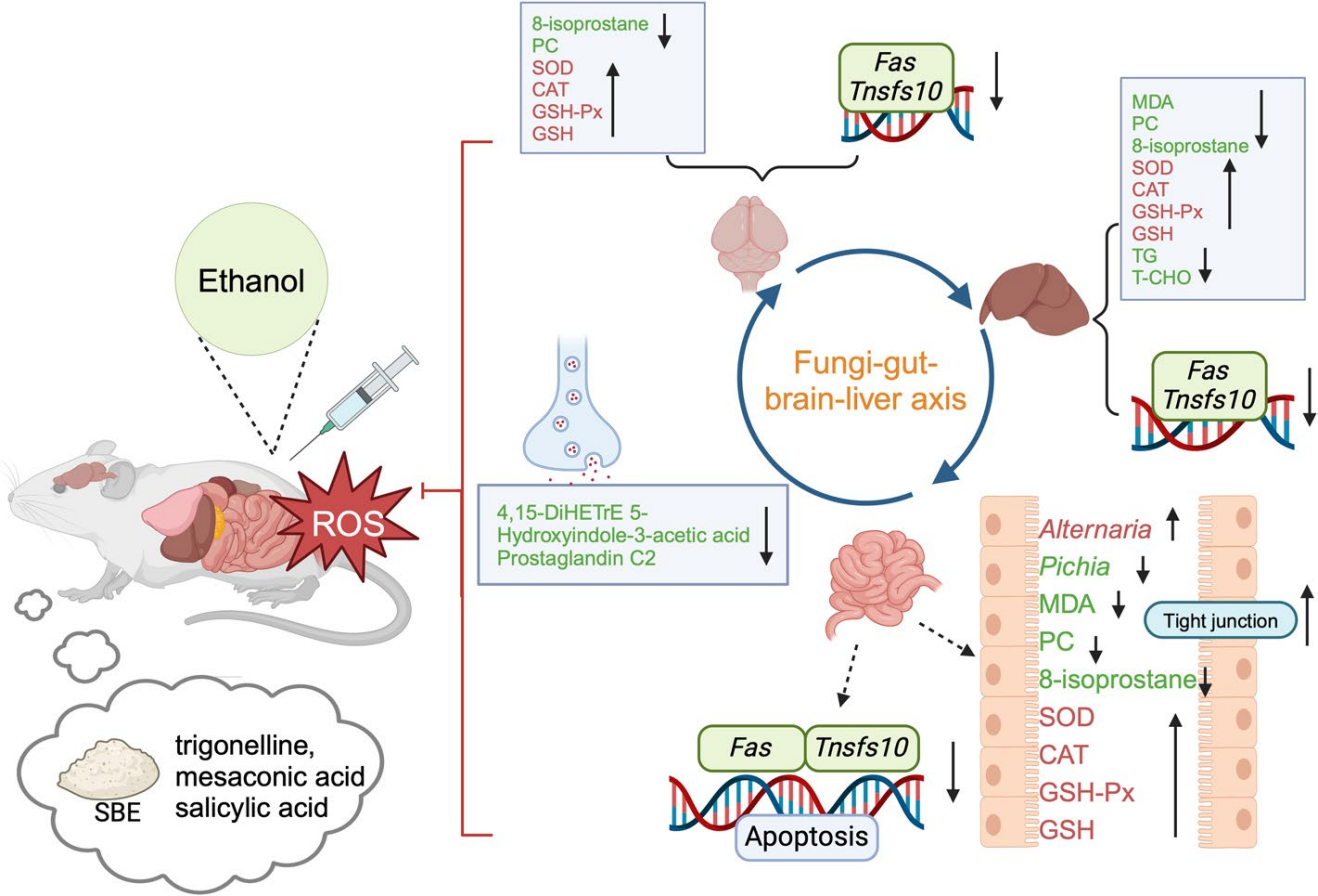

**Supplementary Information:**

The online version contains supplementary material available at 10.1186/s40168-025-02221-8.

## Background

Oxidative stress, a central concept in redox biology and modern medicine [1], not only inflicts damage on macromolecules but also induces cell death and structural tissue damage by disrupting mitochondrial function, activating apoptosis, or promoting necrosis [2, 3]. Emerging evidence has implicated oxidative stress as a direct or indirect contributor to the pathogenesis of various diseases, including inflammatory bowel disease, Alzheimer’s disease (AD), chronic obstructive pulmonary disease, and others [4, 5].

Notably, growing research highlights the critical role of gut microbiota in mediating these diseases [6, 7]. Accumulating research has investigated how natural products [8, 9] modulate the gut microbiota—predominantly bacterial communities, with less focus on fungi [10–13]—to exert mechanisms such as antioxidant stress, thereby ultimately alleviating disease-related functional disorders, metabolic abnormalities, and biomarker abnormalities [11, 14, 15]. For example, the extract of *Vaccinium macrocarpon* Aiton significantly increased the proportion of bacteria *Akkermansia* in oxidative stress mice [16], and exerted beneficial metabolic effects. Copper–luteolin nanocomplexes selectively enrich short-chain fatty acid-producing beneficial bacteria (e.g., *Lachnospiraceae* increased from 12 to 28%, *Bifidobacteriaceae* from 5 to 15%) by mitigating intestinal oxidative stress, while suppressing pro-inflammatory bacteria (e.g., *Enterobacteriaceae* reduced from 25 to 10%) to alleviate oxidative stress [17]. Similarly, blueberry polyphenols alleviates oxidative stress by restoring gut microbiota homeostasis, evidenced by increasing the abundance of bacterial genera (e.g., *Allobaculum* and *Faecalibaculum*) [18].

Furthermore, in recent years, the tripartite interrelation among intestinal microbiota, metabolites, and host genes has emerged as a pivotal research frontier for deciphering the molecular mechanisms underlying physiological homeostasis and pathological transitions [19–23]. Multiple studies have unveiled intricate regulatory networks where metabolic profiles intersect with oxidative stress responses, demonstrating how metabolites modulate redox homeostasis. Metabolites, such as short-chain fatty acids, capsaicin, aspartate, and serotonin, modulate redox homeostasis by enhancing mitochondrial oxidative phosphorylation, promoting β-oxidation, activating AMPK signaling, inhibiting NADPH oxidase activity, and regulating the RIP3-MLKL and RIP1-Nrf2-NF-κB signalling axes, thereby upregulating related gene expression to exert anti-oxidative stress effects [24–28]*.* Those process maintain intestinal microbiota homeostasis, scavenges excessive ROS, modulates the cellular antioxidant response, preserves mitochondrial integrity, and sustains cell viability.

Equally, oxidative stress has an important impact on gene regulation. ROS accumulation induces mitochondrial structural damage (cristae loss and fragmentation), membrane potential dissipation, and cytochrome c release, activating the Nrf2-Keap1-ARE pathway, NF-κB p65 phosphorylation, and apoptotic pathway [29, 30]. For example, the antioxidant *N*-acetylcysteine (NAC) reduces ROS levels and reverses cell death, confirming oxidative stress as a central driver of apoptosis [31]. Additionally, oxidative stress-derived 4-hydroxy-2-nonenal (HNE) can induce *Fas* expression via the c-Jun N-terminal kinase (JNK) pathway, ultimately leading to apoptosis [32]. Moreover, during the differentiation of induced endocardial cushion-like cells, oxidative stress can trigger TET2-mediated 5-hydroxymethylcytosine modification, which activates serotonergic synapse-related genes (e.g., CACNA1F and CYP2D6), thereby promoting vascular maturation and enhancing antioxidant capacity [33]*.*

These findings provide multi-omics evidence for deciphering the regulatory mechanisms of the microbe-metabolite-gene network in oxidative stress, highlight how this multi-level regulatory framework ameliorates oxidative damage, offering new targets and pathways for therapeutic intervention in oxidative stress-associated disorders.

*Sonchus brachyotus* DC. (*S*. *brachyotus*) is widely available as a vegetable and used in traditional folk medicine for removing heat toxins to stop bleeding, intestinal inflammation, and other ailments [34, 35]. Despite its traditional uses, mechanistic studies on *S*. *brachyotus* remain limited. Notable findings include the synergistic alleviation of non-alcoholic fatty liver disease by *S*. *brachyotus* extract in combination with synbiotics [36], and the anti-inflammatory activity of sesquiterpene lactones isolated from this plant [37]. Our previous studies have demonstrated that ethanol-extracted SBE exhibited robust in vitro antioxidant activity [38]. We further reported for the first time that SBE prevented oxidative stress via the Nrf2-Keap1-ARE signal pathway [39], alleviating intestinal oxidative stress through interactions between the oxidative stress biomarkers and gut microbiota [40]. However, the specific mechanisms by which SBE modulates the microbiota-metabolites-genes to alleviate oxidative stress remain unknown. Against this backdrop, the present study aims to explore the mechanism by which SBE exerts antioxidant stress through microbiota-metabolites-genes. Specifically, we constructed a microbiota-metabolites-genes interaction network to elucidate the mechanisms by which SBE mediates its antioxidant effects through bidirectional interactions among microbiota, metabolites, and genes.

## Methods

### Chemicals and reagents

Chemicals and reagents used in this study are listed in Table 1.

**Table 1:** List of chemicals and reagents used in the study

Chemicals and reagents	Company
Total cholesterol assay kit	Nanjing Jiancheng Bioengineering Institute
Triglyceride assay kit	Nanjing Jiancheng Bioengineering Institute
Alcohol dehydrogenase assay kit	Nanjing Jiancheng Bioengineering Institute
Alanine aminotransferase Assay Kit	Nanjing Jiancheng Bioengineering Institute
Aspartate aminotransferase Assay Kit	Nanjing Jiancheng Bioengineering Institute
Low-density lipoprotein cholesterol assay kit	Nanjing Jiancheng Bioengineering Institute
Reduced glutathione (GSH) assay kit	Nanjing Jiancheng Bioengineering Institute
Protein Carbonyl assay kit	Nanjing Jiancheng Bioengineering Institute
Lipid peroxidation MDA assay kit	Beyotime Biotechnology Co., Ltd
Total superoxide dismutase (SOD) assay kit with WST-8	Beyotime Biotechnology Co., Ltd
Catalase (CAT) assay kit	Beyotime Biotechnology Co., Ltd
glutathione peroxidase (GSH-Px) assay kit	Beyotime Biotechnology Co., Ltd
Enhanced BCA protein assay kit	Beyotime Biotechnology Co., Ltd
Mouse 8-isoprostane, ELISA Kit	Wuhan Mercak Biotechnology Co., Ltd
L-012 sodium salt	FUJIFILM Wako Pure Chemical Corporation
E-Cadherin (4A2) Mouse mAb antibody	CST-Cell Signaling Technology
Claudin-1 antibody	Santa Cruz
Goat anti-Mouse IgG (H + L) Highly Cross-Adsorbed Secondary Antibody	Invitrogen
TRIzol®Reagent	Life
TransScript® II Multiplex Probe One-Step qRT-PCR SuperMix UDG	TransGen Biotech
SuperReal PreMix Plus (SYBR Green)	TIANGEN BIOTECH (BEIJING)CO., LTD
Sodium dodecyl sulfate (SDS)	Beijing Biotopped Technology Co., LTD
FAS (G-9) mouse monoclonal antibody	Santa Cruz Biotechnology. Inc
Rabbit Anti-TRAIL antibody (bs-1214R)	Beijing Bioss Biotechnology Co., Ltd
*β-*actin (ACTBD11B7) mouse monoclonal antibody	Santa Cruz Biotechnology. Inc
PageRuler Plus prestain protein Marker	Thermo Scientific
HRP-labeled goat anti-mouse IgG (H+L)	Santa Cruz Biotechnology. Inc

### Extract preparation

The aerial parts of *S*. *brachyotus* DC. were collected from Binzhou City, Shandong Province, China, in 2022. After all samples were dried and crushed to pass through a 60-mesh sieve, and then extracted by ultrasonication for 30 min under the conditions of 75% (v/v) ethanol/water solution, 1:30 (m/v) material to liquid, and 700 ultrasonic power. Subsequently, after centrifugation at 5000 × g for 10 min, the eluate was evaporated by rotary evaporation at 40 ± 2 °C to make a powder out of a liquid plant extract using vacuum freeze-drying technology, which was stored at 4 °C [40].

### Animal experiments

All the described animal experiments in this study performed in the SPF level animal house of the Institute of Chinese Materia Medica, China Academy of Chinese Medical Sciences and complied with the guidelines of Animal Ethics Committee (Ethics No.2022B045).

Adult male mice of SPF grade healthy ICR at 25 ~ 30 g. Animal source: Beijing Viton Lihua Laboratory Animal Technology Co. (Institutional License No.: SCXK (Beijing) 2016–0011). Housing environment: temperature 22 ± 2 °C, 12 h alternating cycle of light and dark, environmental humidity maintained at about 40 ~ 60%. Record the weight of mice using the balance weekly, while also recording their food intake.

The healthy adult mice were randomly divided into five groups, and the handling is shown in Table 2. After 35 days of feeding, the model group (EtOH) and three dose groups (T1, T2, and T3) fasted for 16 h, followed by a single feeding of 50% (v/v) ethanol (food-grade) at 12 mL/kg·BW, and sampling after 6 h. The control group (CON) was sampled without fasting. The principle by which ethanol induces oxidative stress in mice involves: the synergistic effects among multiple pathways (production of the reactive product acetaldehyde; damage to mitochondria; direct or membrane effects caused by hydrophobic ethanol; ethanol-induced hypoxia, etc.) promote ethanol-induced oxidative stress [41]. The acute ethanol model is commonly used to study oxidative stress [42, 43], which is the method for testing and evaluating the Antioxidant Function of Health Food in China (2023 Edition) [44].

**Table 2:** Ethanol-induced acute oxidative stress in adult mice

Group	Feed	Treatment (12 mL/kg·BW)	Quantity per group
CON	Basic diet + drinking water	Water	10
EtOH	Basic diet + drinking water	50% (v/v) ethanol (food-grade)	10
T1	Basic diet + 200 mg/kg SBE	50% (v/v) ethanol (food-grade)	10
T2	Basic diet + 400 mg/kg SBE	50% (v/v) ethanol (food-grade)	10
T3	Basic diet + 600 mg/kg SBE	50% (v/v) ethanol (food-grade)	10

Infraorbital blood was collected into a 1.5 mL centrifuge tube and naturally precipitated, then centrifuged at 3000 rpm for 15 min to enrich the supernatant. Collect the spleen, liver, colon, brain tissues, and cecum contents by dissection, and weigh the spleen and liver quickly and record the weights. Approximately 1 cm of distal the colon and liver lobes were taken and fixed in 4% formaldehyde solution for subsequent histologic analysis. All samples were quickly frozen in liquid nitrogen and stored at − 80 °C for backup.

### In vivo imaging of mice

The luminescent probe L-012 was dissolved in ultrapure water and administered intraperitoneally at a dose of 25 mg/kg in a 100 μL injection volume [45]. During in vivo imaging, mice were anesthetized with 1.5 ~ 2.5% isoflurane and fixed. Mice were executed by cervical dislocation after live imaging, and their organs were rapidly dissected and isolated for imaging. Data acquisition and analysis were performed using the software Living Image and GraphPad Prism 9. Image exposure time was 5 s ~ 2 min, depending on signal intensity.

### Measurement of oxidative stress biomarkers

Peroxidation indicators (MDA, 8-isoprostane, and PC), antioxidant enzymes (CAT, SOD, and GSH-Px), and antioxidants (GSH) were detected in serum, liver, intestinal, and brain tissues according to the kit instructions.

### Histological analysis

Tissues from the mouse colon, liver, and brain were harvested, washed, and fixed with 4% paraformaldehyde, and embedded in paraffin. Paraffin-embedded sections were prepared and stained with H&E for morphological observation [10]. Images were obtained using a Leica microscope. To evaluate the effect of SBE on tissue morphology, three random sections from each organ (colon, liver, and brain) were selected from three mice per group. Morphology was scored as follows: 0 (Normal), 1 (Slight disruption), 2 (Moderate disruption), and 3 (Severe disruption) [46].

### Immunostaining of E-cadherin and Claudin-1

For immunofluorescence staining of intestinal tight junction proteins, colon tissue sections were prepared as follows [47]: colonic tissues were sliced into 4 μm sections, subjected to antigen retrieval in citrate buffer, and then incubated with primary antibodies overnight at 4 °C. The primary antibodies included anti-E-cadherin (14,472, Cell Signaling Technology) and anti-Claudin-1 (sc-166338, Santa Cruz). Alexa Fluor 594 conjugated Affinipure Donkey Anti-Mouse IgG (H + L) (FNSA-0062, FineTest) and Alexa Fluor 488 conjugated Affinipure Goat Anti-Mouse IgG (H + L) (FNSA-0055, FineTest) were used as secondary antibodies. Fluorescence images were acquired using a fluorescence microscope (Zeiss, Oberkochen) under consistent exposure conditions, and mean fluorescence intensity values were analyzed using ImageJ software. At least three mice per group were included in the experiment and analysis.

### 16S rRNA gene amplicon sequencing and fungal internal transcribed spacer region

The extraction of bacterial DNA from intestinal content, along with the sequencing of PCR amplicons and subsequent gene analysis, was conducted by Biomarker Technologies Co., Ltd. in Beijing, China. Utilizing the PacBio sequencing platform, marker genes were sequenced via the single-molecule real-time sequencing (SMRT Cell) method. This was followed by circular consensus sequencing (CCS) to filter sequences, producing Optimization-CCS for clustering operational taxonomic units (OTUs), as well as for species annotation and abundance assessments. Specific barcoded primers were designed based on the 16S full-length primers 27 F (5′-AGRGTTTGATYNTGGCTCAG-3′) and 1492R (5′-TASGGHTACCTTGTTASGACTT-3′), internal transcribed spacer (ITS) full-length primers ITS1F (5′-TASGGHTACCTTGTTASGACTT-3′) and ITS4 (5′-TCCTCCGCTTATTGATATGC-3′). PCR amplification was carried out, and the resulting products were purified, quantified, and normalized to create a sequencing library (SMRT Bell), which was quality-checked prior to being sequenced using the PacBio Sequel. To investigate the differences among samples, significant species differences at the phylum and genus levels were performed [10].

Reads with similarity at the 97.0% level for OTU clustering using Usearch software; intergroup variability was verified using Student’s t-test. Correlation algorithms of Spearman and Pearson were selected. *p* < 0.05 indicates a significant difference, and *p* < 0.01 indicates a highly significant difference.

### Metabolomics sequencing and analysis of mice serum

Samples were prepared according to protocol. Instrumentalesting and data acquisition: Assay data were acquired in positive/negative ion mode. MSe mode under the control of acquisition software MassLynx V4.2 (Waters) was used for primary and secondary mass spectrometry data acquisition on a high-resolution mass spectrometer. LC-QTOF-based qualitative and quantitative metabolomic analyses was performed for the samples. The metabolomic bioinformatics analysis utilized the BMKCloud platform. (www.biocloud.net). Differentially expressed metabolites (DEMs) screening conditions: metabolites with twofold (fold change ≥ 2, FC ≥ 2) or more differential expression between the CON group and EtOH were differentially significant. Based on the above, DEMs were screened by combining the variable importance in projection (VIP) ≥ 1 obtained from the OPLS-DA model of multivariate analysis, and the *p* < 0.05 of the t-test of univariate analysis. The analysis of differential grouping was set as CON vs. EtOH, EtOH vs. T1, EtOH vs. T2, and EtOH vs. T3. Taking CON vs. EtOH differential grouping as an example, CON was used as the control group and EtOH as the experimental group in screening for differentially expressed metabolites.

Heatmap of metabolite expression performed using Microbiotics (https://www.bioinformatics.com.cn), z-score transformation of metabolite expression, clustering method COMPLETE, and distance method Euclidean. Differential metabolite KEGG signaling pathway enrichment: based on the DEMs obtained from screening between the CON group and the EtOH group, using metabolite ID to screen the DEMs between EtOH group and T group, after obtaining the intersecting DEMs, KEGG signaling pathway enrichment analysis was performed, and the results were presented as bubble plots.

### Transcriptomic sequencing and analysis of mice colon tissue

After extraction of nucleic acids from mouse colon using TRIzol, check the nucleic acid concentration using Nanodrop2000 and check the integrity using Agilent, LabChip GX; all the samples’ quality conformed to the library construction. The downstream data were filtered to obtain Clean Data, and the mouse genome as an internal reference (https://www.ncbi.nlm.nih.gov/genome/?term=Mus+musculus) for sequence alignment. Transcriptome bioinformatics analysis of the main BMKCloud platform (www.biocloud.net).

Differentially expressed genes (DEGs) screening conditions: after obtaining all the expressed genes, the software DESeq2_EBSeq was further utilized to set the gene expression differential folds FC ≥ 1.5 with the FDR ≤ 0.01 to screen the DEGs.

### Multi-omics analysis

The combined microbiome, transcriptomics, and metabolomics were analyzed. The genus-level abundance of microbiome significantly associated with markers of oxidative stress, DEGs (FC ≥ 1.5, FDR ≤ 0.01) obtained by transcriptomics, and DEMs (FC ≥ 2, *P* < 0.01) screened by metabolomics, with correlation filtering microbial threshold of 0.7 and correlation *p* < 0.05, were respectively subjected to Spearman. After the correlation analysis data were obtained, the correlation results were plotted using Cytoscape and presented as a correlation network diagram.

### Quantitative reel time PCR

Tissues of intestine, liver, and brain around 1 cm^3^ in diameter were ground by liquid nitrogen, added with 1 mL of Trizol reagent to extract total RNA, and tested for RNA concentration and purity by an enzyme labeling instrument. Reverse transcription using cDNA reverse transcription kit to obtain cDNA. SuperReal PreMix Plus (SYBR Green) was used for real-time quantitative PCR (qRT-PCR). Design the qRT-PCR primer sequences using the NCBI (https://www.ncbi.nlm.nih.gov) website [39, 45]. Gene expression was normalized to the expression of *β-Actin*. qRt-PCR primer sequences are shown in Table 3.

**Table 3:** Sequences of qRT-PCR primers

*Gene*		Primer Sequence (5′−3′)
*Rab19*	F	CTACAGTGAGTCCCAGCAGAAC
	R	CCCACACCTGCATCTTCACT
*Zfand2a*	F	CTCTGACTACCCGTGATACTTAAA
	R	GCATGTTATTGGCAGAAAATCTAGC
*C2cd4b*	F	ACTACACGTCACCTGCTTCG
	R	GCGGATACACGAGCGTCTTTT
*Etv4*	F	CAGACTTCGCCTACGACTCA-
	R	GCCATAACCCATCACTCCAT
*Rpl21*	F	GTGGGGAAGGAAGTAACTCGC
	R	TGAACAGTGCCCATTCCCTT
*Rasef*	F	AGGAGATCTGGAGTTACGGTGAA
	R	ACACGACTAAGCAGCCACAAT
*Fas*	F	GTCAACCATGCCAACCTGAAAAC
	R	CACCCCCTGCAATTTCCGTT
*Tnfsf10*	F	GCCAGCTCTGCTGTTTTGAG
	R	CACCTGGTGGCACTAATGGT
*β-Actin*	F	TGTCCACCTTCCAGCAGATGT
	R	AGCTCAGTAACAGTCCGCCTAGA

### Western blot

For sample preparation, utilized the lysis buffer to break down the intestinal, liver, and brain tissues. Mixed the cell suspension intermittently, then gathered the supernatant and centrifuge it at 12,000 rpm for 15 min at 4 °C, and then measured the content of protein using the BSA kit. PBS was used to adjust the total sample volume to achieve a final protein concentration of 1 µg/mL. After preparing the 10% (w/v) SDS-PAGE separation gel and concentrate gel, the samples were mixed with loading buffer and separated in an orderly manner. The protein was transferred to a PVDF membrane, which was blocked with a 5% skimmed milk powder buffer at room temperature for 2 h, then incubathe membranes incubatinged with the corresponding primary antibodies (Fas, Tnsfs10, and β*-*Actin) overnight at 4 °C. Following this, the samples were then rinsed with 1 × TBST three times, incubated with a secondary antibody for 2 h at room temperature, and then PVDF rinsed with 1 × TBST three times. To visualize the protein bands, the PVDF membrane was placed flat in the LI-COR Odyssey CLx imaging system, and the protein bands were measured with Image Studio™ Software, and the values of the protein bands to be measured were compared with the internal reference protein to calculate the relative expression of the targets [39].

### LC–MS/MS analysis

A Thermo Fisher Orbitrap Exploris 120 (O-Exative) mass spectrometer was used for SBE identification and Xcalibur software is employed to accurately extract ion peaks for compound identification based on precise m/z values. During analysis, 2 μL sample was injected through ACQUITY UPLC HSS T3 (2.1 × 100 mm, 1.8 µm) with a gradient mobile phase consisting of ACN (solvent A) and 0.1% (v/v) formic acid solution (solvent B) at a flow rate of 0.3 mL/min. The Elution gradient program was as follows: 0 ~ 24 min, 5 ~ 100% A; 24 ~ 26.5 min. 100% A; 26.5 ~ 27 min. 100 ~ 5% A. 27 ~ 30 min, 5% A. For mass detection, a heated electrospray ionization (HESI) source was operated in positive and negative mode with a scan range from 100 ~ 1500 Da, and data were collected in Full-ms/dd-MS.

### Acquisition of targets of active ingredients

The targets of active ingredients were obtained or predicted by the PubChem (https://pubchem.ncbi.nlm.nih.gov), a bioinformatics analysis tool for molecular mechanism of Traditional Chinese Medicine (BATMAN-TCM, http://bionet.ncpsb.org.cn/batman-tcm/#/home), SwissTargetPrediction (http://www.swisstargetprediction.ch), and ChEMBL (https://www.ebi.ac.uk/chembl/) databases, and then the targets of active ingredients were obtained by gene name conversion using uniProt database.

### Molecular docking

The 3D structures of chemical compounds were retrieved from the PubChem database. Then, we obtained the structures of target proteins Fas and Tnfsf10 in .pdb format from the databases of UniProt and PDB. In addition, OpenBable 2.4.1 and PyMOL 2.2.0 were used to pre-process the pharmacophore and target proteins through hydrogenation, dehydrogenation, and decharging, and then the processed pharmacophore molecules were molecularly docking with the target proteins suing AutoDock 4.2.6 software. Finally, the results were visualized by PyMOL 2.2.0 software.

#### Data and statistical analysis

Continuous data were expressed as mean ± standard error of the mean (SEM). Statistical significance was defined as *p* < 0.05 (significant) or *p* < 0.01 (highly significant). For data involving more than two groups, one-way analysis of variance (ANOVA) followed by Duncan’s post-hoc test was used. Comparisons between the two groups were analyzed using Student’s t*-*test. Analyses of microbial diversity, differential metabolites, and differentially expressed genes were conducted using BMKCloud (www.biocloud.net accessed on 1 December 2022). Correlation analyses among microbiome, metabolome, and transcriptome datasets were performed using the Spearman algorithm with a threshold of |r| > 0.7 and *p* < 0.05. Details of the specific statistical tests, sample sizes, and replicate numbers for each experiment are provided in the corresponding figure legends. All data shown are representative of at least three independent experiments [46, 48].

## Results

### Effect of SBE on oxidative stress biomarkers in serum, intestine, liver, and brain tissues

**Fig. 1:**
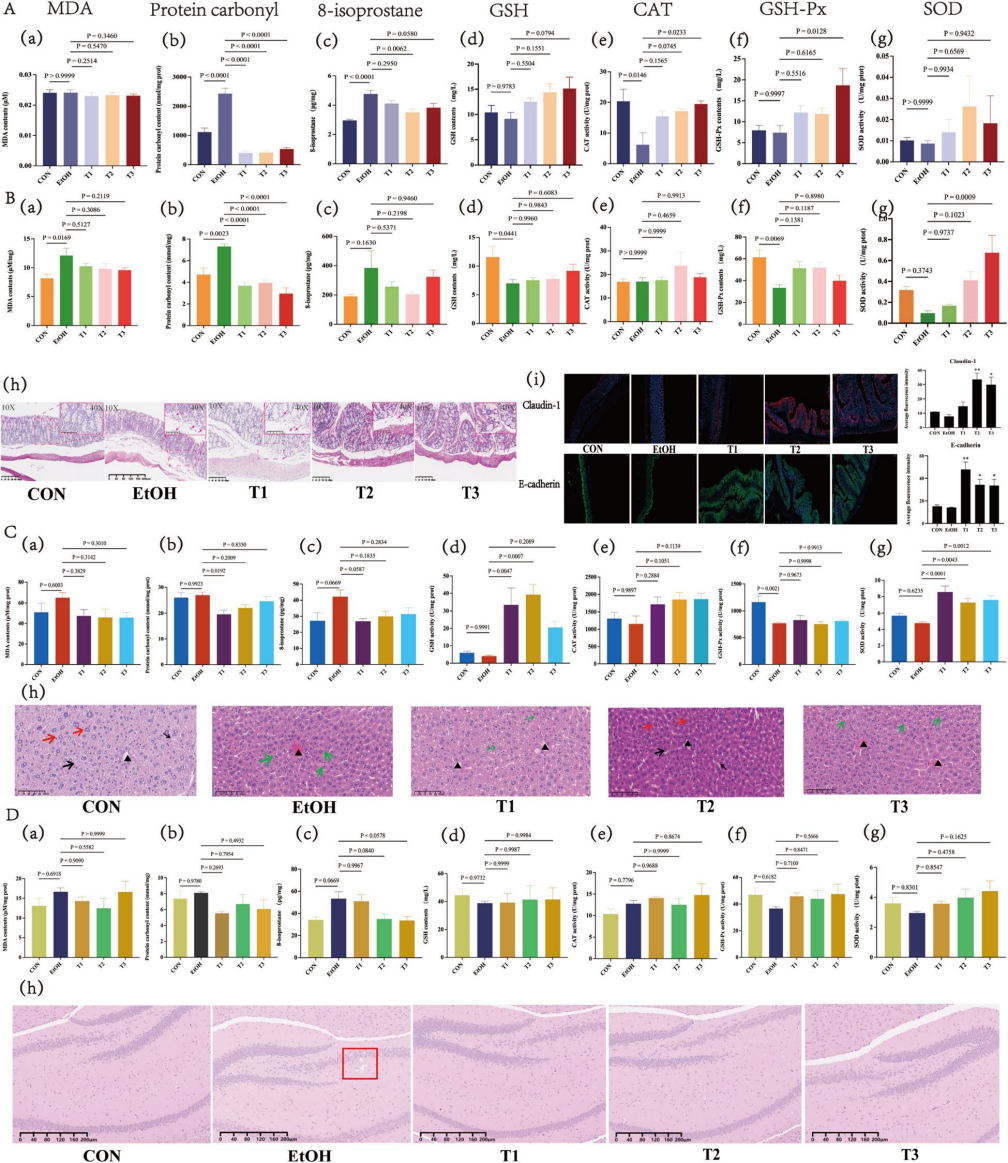
Effect of SBE on oxidative stress biomarkers. Effect of SBE on oxidative stress biomarkers in serum (*n* = 6) (**A**). The changes of MDA, 8-isoprostane, PC and GSH contents in the serum of different groups’ mice (a–d). The changes of CAT, GSH-Px, and SOD activities in the serum of different groups’ mice (e–g). Effect of SBE on oxidative stress biomarkers in intestinal tissue (*n* = 6) (**B**). The changes of MDA, 8-isoprostane, PC, and GSH contents in the colon tissue of different groups’ mice (a–d). The changes of CAT, GSH-Px, and SOD activities in the colon tissue of different groups’ mice (e–g). H&E staining analysis of intestinal tissue morphology. Arrows indicate inflammatory cell infiltration (*n* = 3) (h). Immunofluorescence analysis of the expression levels of E-cadherin and Claudin-1 tight junction protein (*n* = 3). Compared with the EtOH group, *p < 0.05, **p < 0.01 (i). Effect of SBE on oxidative stress biomarkers in liver (**C**). The changes of MDA, 8-isoprostane, PC, and GSH in the liver tissue of different groups of mice (a–d). The changes of CAT, GSH-Px, and SOD activities in the liver tissue of different groups’ mice (e–g). H&E staining analysis of the liver tissue morphology (*n* = 3). Thin arrows indicate goblet cells. Thick arrows indicate eosinophils. Hepatocytes: thin red arrow; Central vein: black triangle; Hepatic sinus: thin black arrow; Bubble: Green arrow; Pink-stained particles: thick red arrows (h). Effect of SBE on oxidative stress biomarkers in brain tissue (*n* = 6) (**D**). The changes of MDA, 8-isoprostane, PC, and GSH contents in the brain tissue of different groups’ mice (a–d). The changes of CAT, GSH-Px, and SOD activities in the brain tissue of different groups’ mice (e–g). H&E staining analysis of brain tissue morphology (*n* = 3). Red box: Hippocampal neuronal degeneration (h). Data are the means ± SEM

MDA, 8-isoprostane, and PC serve as key biomarkers of lipid and protein peroxidation, reflecting the rate and intensity of peroxidation in vivo. The antioxidant defense system comprises enzymatic antioxidants like SOD, GSH-Px, CAT, and non-enzymatic antioxidants like GSH, which collectively scavenge H_2_O_2_ and free radicals. We investigated the impact of SBE on these biomarkers in serum, as well as in the intestine, liver, and brain tissues of mice (Fig. 1).

Compared with the CON group, the EtOH group exhibited significantly elevated levels of PC and 8-isoprostane in serum, as well as MDA and PC in intestinal tissue (*p* < 0.05). Concomitantly, EtOH exposure reduced GSH content in the intestine, alongside decreased CAT activity in serum and GSH-Px activity in both intestinal and hepatic tissues (*p* < 0.05). SBE treatment at different doses produced distinct regulatory effects. The T1 group showed significant reductions in serum, intestinal, and hepatic PC levels, accompanied by increased GSH content in the intestine and liver, as well as enhanced SOD activity in the liver (*p* < 0.05). The T2 group significantly decreased serum PC and 8-isoprostane levels, along with intestinal PC levels, while elevating hepatic GSH content and hepatic SOD activity (*p* < 0.05). The T3 group demonstrated reduced serum and intestinal PC levels, coupled with increased serum CAT and GSH-Px activities, as well as intestinal and hepatic SOD activities (*p* < 0.05). Other measured indices showed no significant differences from the EtOH group. All SBE-treated groups exhibited a trend toward restoring these biomarkers to CON levels (Fig. 1).

The colonic tissue of the CON group exhibited normal architecture, whereas the model group showed mild inflammatory cell infiltration in the lamina propria and submucosa. Treatment with SBE reduced the degree of inflammatory infiltration in a dose-dependent manner, with the highest dose (T3) demonstrating the most pronounced efficacy (Fig. 1B(h)). Immunofluorescence staining revealed downregulated expression of tight junction proteins Claudin-1 and E-cadherin in the EtOH group, which was significantly restored by SBE treatment (*p* < 0.05) (Fig. 1B(i)). H&E staining of liver tissue showed diffuse cytoplasmic vacuolization and lipid degeneration in EtOH-treated mice. And, the T1 group showed no significant improvement, whereas both T2 and T3 groups normalised hepatic morphology (Fig. 1C(h); Supplementary Material Result 3). In brain tissue, the EtOH group exhibited hippocampal neuronal degeneration, which was fully reversed in all SBE-treated groups, with no evidence of apoptosis observed across any group (Fig. 1D(h)). Although histological changes were evident, oxidative stress markers in brain tissue did not show significant fluctuations. Collectively, these findings indicate that SBE ameliorates oxidative stress by modulating the levels of MDA, 8-isoprostane, PC, CAT, GSH, GSH-Px, and SOD across multiple tissues.

### Effect of SBE on gut microbiota

**Fig. 2:**
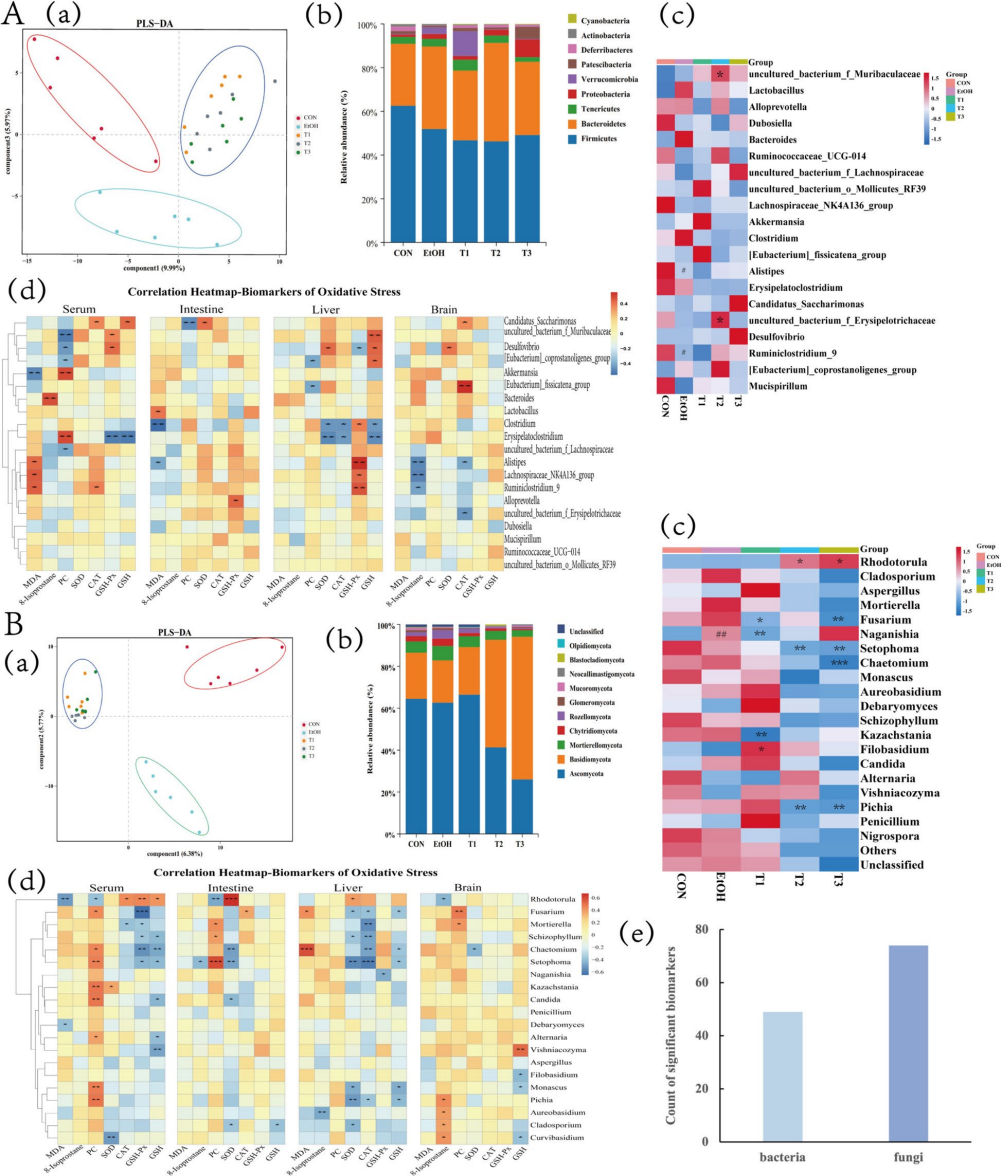
Correlation analysis of gut microbiota and oxidative stress biomarkers. Gut bacteria (*n* = 6) (**A**). Gut bacterial PLS-DA for beta diversity analysis (a). Bacterial species composition at the phylum level (b). Heat map clustering and significant difference analysis on the relative abundance of the top 20 bacterial species at the genus level. Compared with the CON group, ^#^*p* < 0.05. Compared with the EtOH group, *p < 0.05 (c). Correlation analysis between gut bacteria and oxidative stress biomarkers. The redder the color represents a positive correlation and the bluer the color represents a negative correlation. *p < 0.05, **p < 0.01 (d). Gut fungi (*n* = 6) (**B**). Gut fungi PLS-DA for beta diversity analysis (a). Fungi species composition at the phylum level (b). Heat-map clustering and significant difference analysis on the relative abundance of the top 20 fungi species at the genus level. Compared with the CON group, ^#^*p* < 0.05, compared with the EtOH group, *p < 0.05 (c). Correlation analysis of between gut fungi and oxidative stress biomarkers. The redder the color represents a positive correlation, and the bluer the color represents a negative correlation. *p < 0.05, **p < 0.01, ***p < 0.001 (d). Counts of the significant associations between bacteria or fungi and oxidative stress biomarkers. Light blue represents bacteria, dark blue represents fungi (e)

To explore the relationship between oxidative stress biomarkers and gut microbiota, we analyzed the composition of caecal bacteria and fungi. Notable differences were observed in the levels of phylum and genus of bacteria and fungi among CON, EtOH, and T groups (Fig. 2A, B(a)). At the phylum-level, *Firmicutes*, *Bacteroidetes*, *Proteobacteria*, and *Tenericutes* were the predominant bacterial phyla, with *Firmicutes* and *Bacteroidetes* being the most abundant (Fig. 2A(b)). At the genus level, the EtOH group showed significantly downregulated relative abundance of *Alistipes* and *Ruminiclostridium*_*9* (*p* < 0.05), which were restored in T groups. The T2 group exhibited up-regulated relative abundance of *uncultured*_*bacterium*_*f*_*Erysipelotrichaceae* and *uncultured*_*bacterium*_*f*_*Muribaculacea* compared to the EtOH group (*p* < 0.05) (Fig. 2A(c)).

Correlation analysis showed that *Candidatus*_*Saccharimonas* was positively correlated with the SOD/CAT activities and GSH content, but negatively correlated with the PC contents in serum, intestine, and brain tissues. *Uncultured*_*bacterium_f_Muribaculaceae*, *Desulfovibrio*, [*Eubacterium*]_*coprostanoligenes*_*group*, and [*Eubacterium*]_*fissicatena*_*group* showed negative correlations with the PC contents, but positive correlations with the GSH contents and CAT activities in serum, liver, and brain tissues. *Clostridium* suggested negative correlations with the GSH content, CAT/SOD activities in liver tissue, while *Erysipelatoclostridium* negatively correlated with the GSH-Px/SOD/CAT activities and GSH contents, but positively with the PC contents in serum and liver tissue. *Alistipes*, *Lachnospiraceae*_*NK4A136*_*group*, and *Ruminiclostridium*_*9* positively correlated with the liver GSH-Px activity and negatively with the brain 8-isoprostane contents.

For fungi, phylum-level analysis showed dominance of *Ascomycota, Basidiomycota*,* Mortierellomycota*, and *Chytridiomycota* (Fig. 2B(b)). Compared to the EtOH group, the T2/T3 groups have downregulated the relative abundance of *Setophoma* and *Pichia* (*p* < 0.01) and the relative abundance of *Rhodotorula* (*p* < 0.05), while the T3 group down-regulated the relative abundance of *Chaetomium* and *Fusarium* (*p* < 0.05). T1 group upregulated the relative abundance of *Filobasidium* (*p* < 0.05) and downregulated relative abundances of *Fusarium*, *Kazachstania,* and *Naganishia* (*p* < 0.01). The EtOH group upregulated relative abundance of *Naganishia* (Fig. 2B(c)).

*Rhodotorula* positively correlated with serum SOD/GSH-Px activities and GSH content (*p* < 0.05), but negatively with serum/liver 8-isoprostane and PC contents (*p* < 0.05). *Fusarium* and *Mortierella* positively correlated with brain PC content (*p* < 0.05), but negatively with liver CAT activities (*p* < 0.05). *Schizophyllum*, *Chaetomium*, and *Setophoma* negatively correlated with liver CAT activities (*p* < 0.05), while *Mortierella*, *Schizophyllum*, and *Setophoma* positively correlated with intestine PC contents (*p* < 0.05). *Alternaria* positively correlated with serum PC contents, but negatively with serum GSH contents (*p* < 0.05). *Monascus* negatively correlated with liver SOD activities and GSH contents, but positively with serum PC contents. *Pichia* positively correlated with serum/brain 8-isoprostane and PC contents (*p* < 0.05), and negatively with liver SOD, CAT, and GSH. *Aureobasidium*, *Cladosporium*, and *Curvibasidium* positively correlated with brain 8-isoprostane (*p* < 0.05).

These results suggest SBE modulates gut fungal/bacterial abundance to regulate SOD/GSH-Px/CAT activities and MDA/8-isoprostane/PC/GSH levels. Notably, 49 bacteria and 74 fungi showed strong associations with oxidative stress biomarkers (Fig. 2B(e)), indicating a potentially greater fungal contribution to oxidative stress regulation.

### Effect of SBE on serum metabolite

**Fig. 3:**
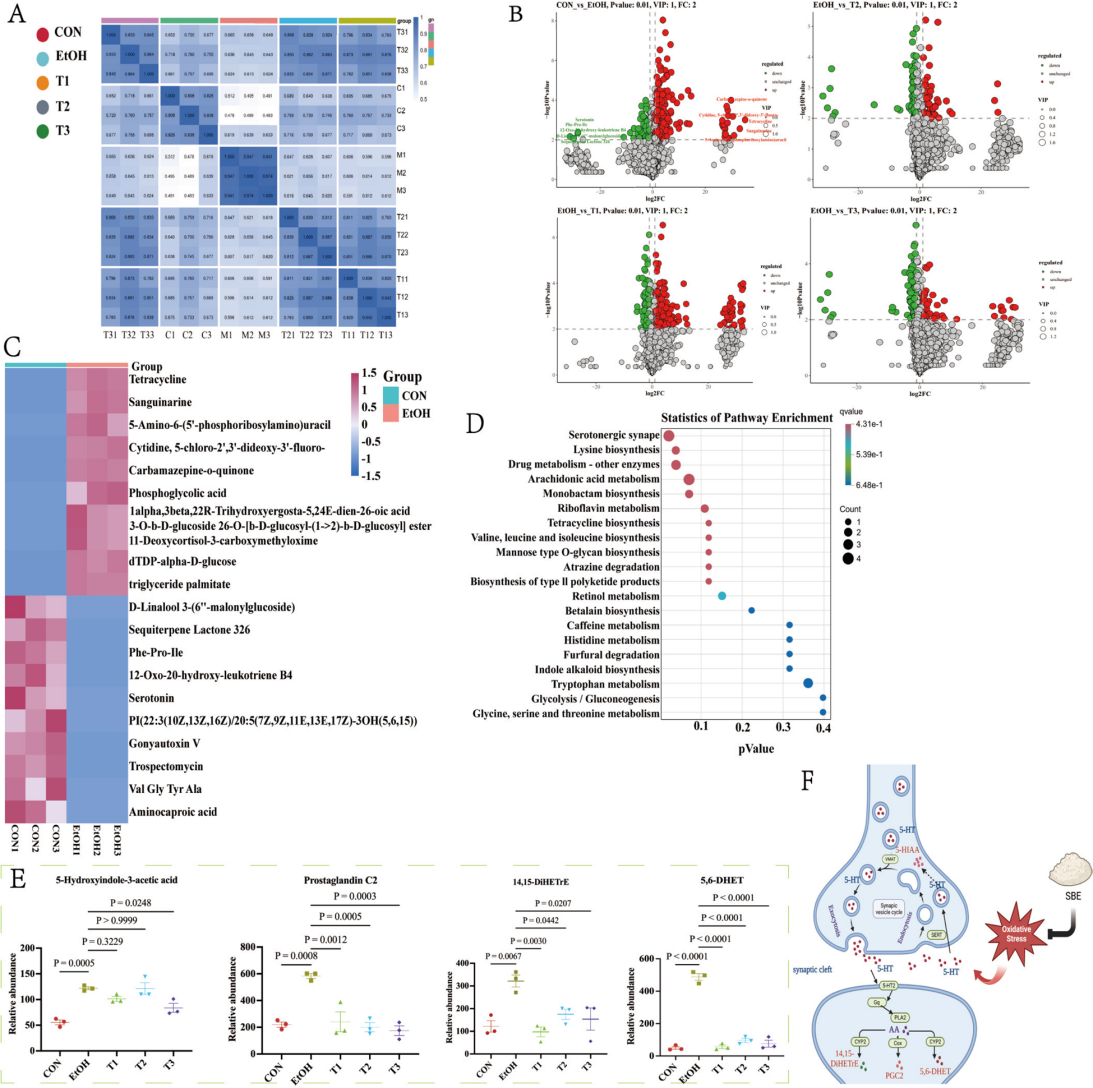
Effect of SBE on the serum metabolites. Repetitive correlation of metabolomics data. The heat map illustrates data distribution across different groups (CON, EtOH, T1, T2, T3). The square of the Spearman Rank Correlation coefficient (*r*^2^) was used as the evaluation index for the correlation of biological replicates. The closer *r*^2^ is to 1, the stronger the correlation between the two replicate samples. Both the horizontal and vertical coordinates represent sample names, the color depth indicates the correlation coefficient, and “Group” represents the grouping (**A**). Differential metabolite volcano maps of differential metabolites for pairwise comparison. CON vs. EtOH (top—left), EtOH vs. T2 (top—right), EtOH vs. T1 (bottom—left), and EtOH vs. T3 (bottom—right). The x-axis represents log₂ (fold change, FC), and the y-axis represents—log₁₀ (*P*-value). Dots above the dashed lines meet the criteria of *p*-value ≤ 0.01, VIP ≥ 1, and FC ≥ 2. Green dots indicate downregulated metabolites, red dots indicate upregulated metabolites, and gray dots indicate non-significantly regulated metabolites. VIP (variable importance in projection) values are visualized by dot size (**B**). Heat map of the top 20 up-DEMs and down-DEMs between CON vs. EtOH. The x-axis represents individual samples, and the y-axis shows the Z-score standardized quantitative values of metabolites after hierarchical clustering. The color bar at the top labels and distinguishes different groups (**C**). KEGG signal pathway enrichment bubble plot. The x-axis represents the *p* value, indicating the statistical significance of pathway enrichment. The y-axis lists the enriched metabolic or biological pathways. Dot color corresponds to the *q* value, with the color scale shown on the top right. Dot size is determined by the “Count”, representing the number of differentially expressed metabolites involved in each pathway (as defined by the size legend on the middle right) (**D**). The relative abundance results of key metabolites of serotonergic synapse pathway (**E**). The potential mechanism diagram of SBE ameliorates oxidative stress by regulating serotonergic synaptic metabolic pathways (Created in BioRender. Tu, D. (2025) https://BioRender.com/883v35c) (**F**). Data are the means ± SEM (*n* = 3)

We conducted metabolomic analysis to characterize the alterations in serum metabolic profile induced by ethanol exposure and SBE treatment. Across positive and negative ion modes, a total of 13,183 peaks were detected, and 4125 metabolites annotated. Intrasample correlation analysis revealed high biological repeatability, validating the reliability of differential metabolite identification and the stability of model prediction (Fig. 3A). Untargeted metabolomics identified 387 significantly altered metabolites (*p* < 0.05) in EtOH vs. CON, including 314 upregulated and 73 downregulated metabolites (Supplementary Table 6). Volcano plots demonstrating distinct metabolic profile separations, both upregulated and downregulated DEMs were observed between CON vs. EtOH and T vs. EtOH groups (Fig. 3B). For the top 20 DEMs, we performed clustering analysis, followed by functional annotation. The results revealed that ethanol exposure primarily altered metabolites from classes such as carboxylic acids and derivatives, organooxygen compounds, fatty acyls, lactones, and others (*p* < 0.05) (Fig. 3C).

To explore the regulatory mechanisms of SBE, we performed KEGG enrichment analysis on the intersecting DEMs across the CON, EtOH, and T groups. KEGG enrichment analysis identified the serotonergic synapse, lysine biosynthesis, drug metabolism, and arachidonic acid metabolism as key pathways, with the serotonergic synapse pathway showing the highest enrichment significance (Fig. 3D). Statistical analysis of serotonergic synapse-related DEMs revealed that key metabolites including 5-hydroxyindole-3-acetic acid, 14,15-DiHETrE, prostaglandin C2, and 5,6-DHET were markedly upregulated in EtOH groups, whereas SBE intervention significantly downregulated these metabolites (*p* < 0.05) (Fig. 3E, Supplementary Table 7). Collectively, these findings indicate that SBE ameliorates oxidative stress by modulating serotonergic synapse pathway metabolites (5-hydroxyindole-3-acetic acid, 14,15-DiHETrE, prostaglandin C2, and 5,6-DHET) (Fig. 3E).

### Effects of SBE on colon genes expression

To investigate how SBE exerts its anti-oxidative stress effects by regulating intestinal gene expression, we employed high-throughput transcriptome sequencing (RNA-Seq). This technique was employed to compare the mRNA expression profiles of intestinal tissues among mice from the CON, EtOH, and T groups; identify DEGs; and explore their associated signalling pathways and potential molecular targets. Transcriptomic analysis of 30 samples generated 384.79 Gb of Clean Data (an average of 5.90 Gb per sample), with Q30 base percentages of at least 94.53%. Clean Reads were aligned to the reference genome, yielding alignment efficiencies rangeing from 85.26 to 96.12%. Subsequent analyses identified 9176 novel genes, of which 2067 were annotated.

**Fig. 4:**
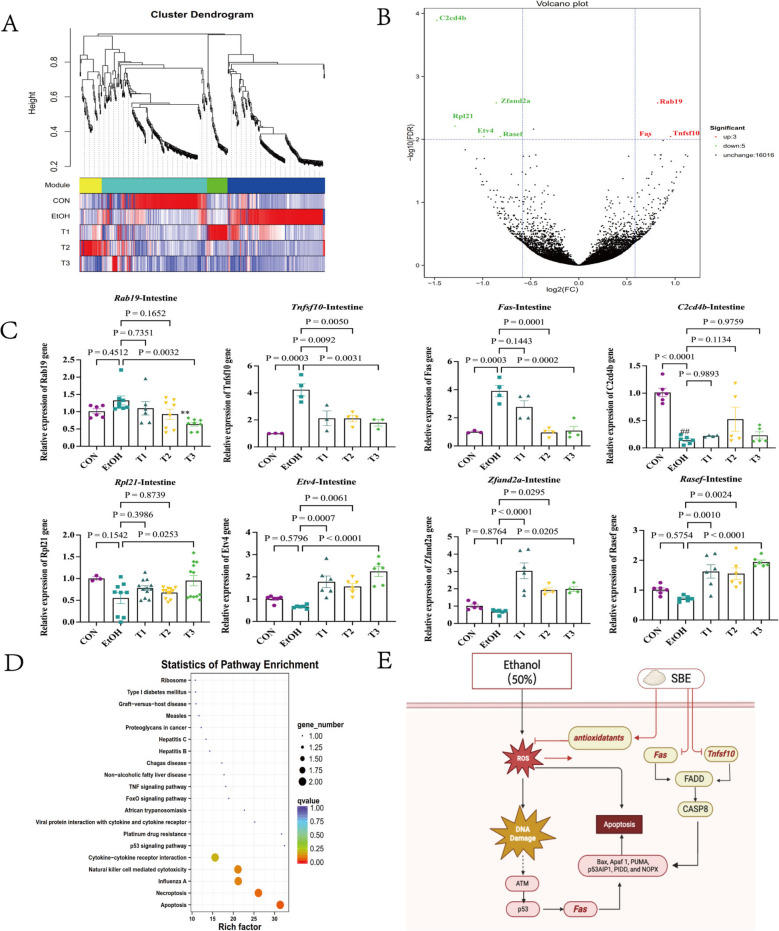
Effect of SBE on the mRNA expression level of genes in the colon. Hierarchical cluster tree showing co-expression modules based on WGCNA analysis. Every leaf on the tree represents a single gene, while the branches represent clusters of closely connected genes. Each is denoted by branches of different colors. The colored rows beneath the dendrograms indicate the module membership of mouse genes (*n* = 6) (**A**). Volcano plot of the DEGs of CON vs. EtOH. The green dots represent down-regulated DEGs, the red dots represent up-regulated DEGs, and the black dots represent non-DEGs (*n* = 6) (**B**). The graph of the relative expression levels of DEGs. Compared with the CON group, ^*##*^*p* < 0.01; compared with the EtOH group, **p < 0.01 (*n* = 6) (**C**). The diagram of KEGG signal pathway enrichment (**D**). The potential mechanism diagram of SBE ameliorates oxidative stress by regulating the apoptosis signaling pathway (Created in BioRender. Tu, D. (2025) https://BioRender.com/h87aqzi) (**E**). Data are the means ± SEM

To explore modules weighted associated with treatment groups, we performed weighted gene co-expression network analysis (WGCNA). The CON group correlated with the light green module, EtOH with the blue module, T1 with the green module, T2 with the yellow module, and T3 partially overlapped with the light green module (Fig. 4A), all of those revealed DEGs across groups. Using DESeq2_EBSeq (fold change ≥ 1.5, FDR < 0.01), we identified 8 DEGs between CON and EtOH: 3 upregulated genes (*Tnfsf10*, *Rab19, Fas*) and 5 downregulated genes (*C2cd4b*, *Rpl21*, *Etv4, Zfand2a*, *Rasef*). Among these, *Tnfsf10* showed the highest fold change, *Rab19* had the most significant difference, and *C2cd4b* exhibited the greatest downregulation (Fig. 4B). qRT-PCR validation confirmed that EtOH upregulated the expression of *Tnfsf10* and *Fas* (*p* < 0.05), while downregulating the expression of *C2cd4b* (*p* < 0.05). KEGG enrichment analysis of DEGs highlighted apoptosis, necroptosis, and cytokine signaling pathways, with the apoptotic pathway being the most significantly enriched. This pathway involved key genes *Tnfsf10* and *Fas* (Fig. 4D). Collectively, these findings suggest that SBE modulates oxidative stress via the apoptosis signaling pathway by modulating the expression of *Tnfsf10* and *Fas* (Fig. 4E).

### SBE exerts its anti-oxidative stress effect by modulating the microbiota-metabolite-gene through its key active components—trigonelline, mesaconic acid, and salicylic acid

**Fig. 5:**
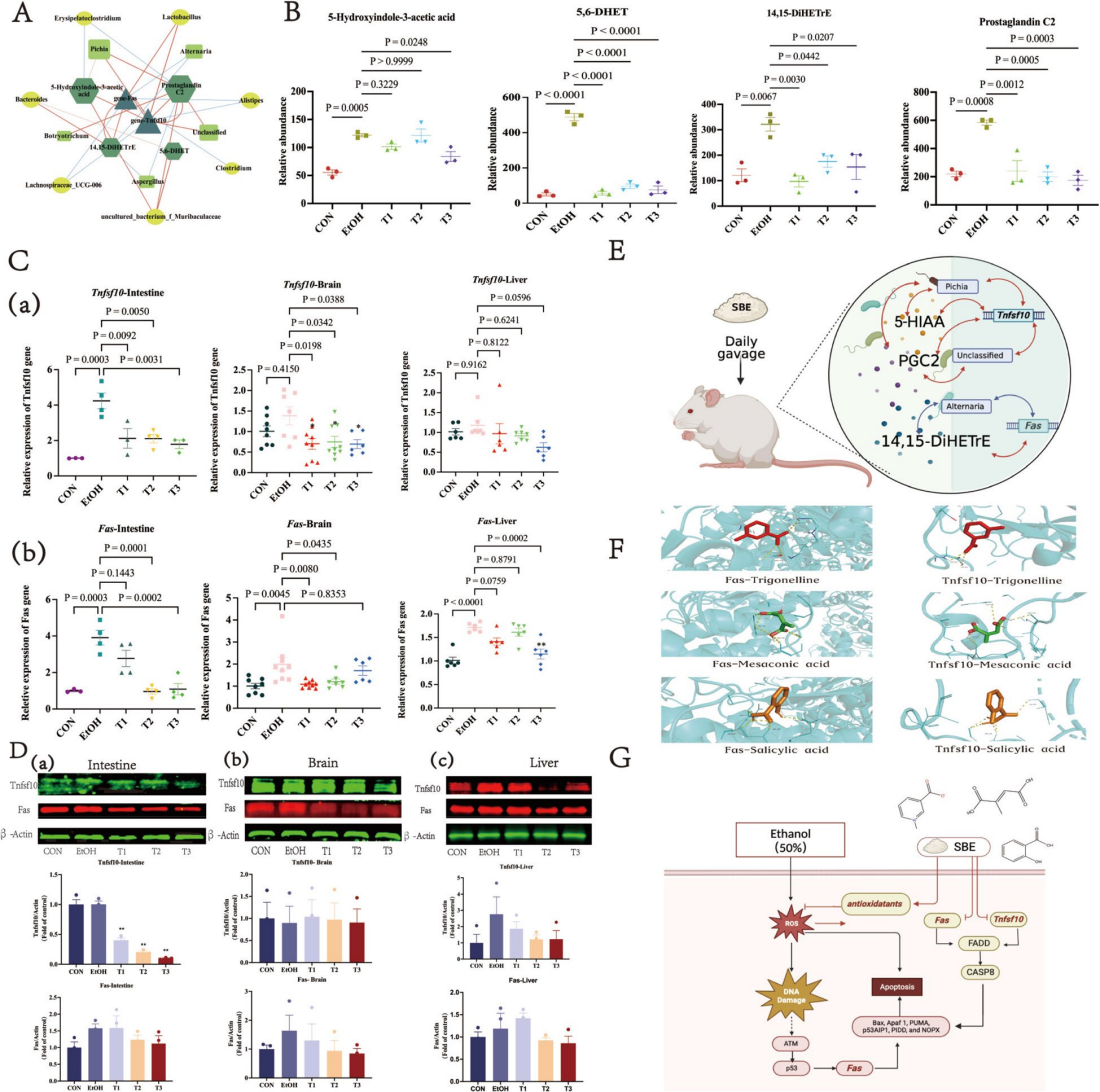
SBE plays an anti-oxidative stress role by regulating the fungi-metabolites-gene through its key active components—trigonelline, mesaconic acid, and salicylic acid. The conjoint analysis results of microbiota, transcriptomics, and metabolomics. Yellow circles represent bacteria, light green rectangles represent fungi, emerald green hexagons represent metabolites, and dark green triangles represent genes. The red line represents positive correlation, the blue line represents negative correlation, and the thickness of the line represents the size of the correlation coefficient (**A**). Graph of intergroup relative abundance results of key metabolites of the serotonergic synapse pathway (*n* = 3) (**B**). qPCR validation of key genes in intestine, brain, and liver tissues. (*n* ≥ 3) (**C**). The protein expression of Tnfsf10 and Fas in the intestine, brain, and liver tissues by Western blot and quantitation using Image Studio software. Compared with the EtOH group, *p < 0.05. (*n* = 3) (**D**). SBE plays an anti-oxidative stress role by regulating the fungi-metabolites-gene. The red arrow represents positive regulation, and the blue arrow represents negative regulation (Created in BioRender. Tu, D. (2025) https://BioRender.com/cposo8c) (**E**). Structure–activity relationship analysis from molecular docking. Light blue represents receptor target proteins. Red, green, and brown denote ligand small molecules trigonelline, mesaconic acid, and salicylic acid, respectively. Dark blue indicates amino acid residues, yellow indicates hydrogen bonds, and values indicate amino acid residue sites (**F**). The key compounds of SBE exhibited antioxidants that could act on both the key targets of Fas and Tnfsf10 in the apoptosis signaling pathway (**G**). Data are the means ± SEM

A number of previous studies have highlighted the intricate relationships among microorganisms, metabolites, and genes. Here, we employed Spearman correlation analysis to characterize these interconnections, aiming to elucidate the antioxidant mechanism of SBE through the microbiota-metabolite-gene. We systematically evaluated the interplay among microbiota, metabolites and genes by integrating prescreened data: 8 DEGs (*Tnfsf10*, *Rab19*, *Fas*, *C2cd4b*, *Rpl21*, *Etv4*, *Zfand2a*, *Rasef*) (Fig. 4), four pivotal metabolites (14,15-DiHETrE, 5-hydroxyindole-3-acetic acid, prostaglandin C2, 5,6-DHET) of the serotonergic synaptic signaling pathway (Fig. 3) and the Top 20 enriched bacterial/fungal genera (Fig. 2). Correlation analysis revealed significant associations (*p* < 0.05) across these components (Supplementary Table 9). Using Cytoscape, we visualized the fungi and bacteria associated with the key gene of the apoptotic signaling pathway and the metabolites of the serotonergic synaptic pathway (Fig. 5A).

Specifically, *Alternaria* negatively correlated with the level of 14,15-DiHETrE and the expression level of *Fas*, while the expression level of *Fas* positively correlated with the expression level of 14,15-DiHETrE. *Pichia* showed positive correlations with the expression level of T*nfsf10* and the level of 5-HIAA/prostaglandin C2, and T*nfsf10* showed positive correlations with the expression level of 5-HIAA/prostaglandin C2 (Fig. 5A). We further validated the identity of the 14,15-DiHETrE, 5-HIAA, and prostaglandin C2 via chemical standard-based analysis (Fig. 5B). Transcriptional and translational analysis showed the levels of *Fas* and *Tnfsf10* expression were notably elevated in the EtOH group than CON and T groups at both levels, across intestinal, brain, and liver tissues (Fig. 5C, D).

Based on the constructed correlation network among metabolites, genes, and fungi, we proposed a hypothetical model depicting how SBE exerts antioxidant effects via the fungi-metabolite-gene (Fig. 5E). SBE modulates the relative abundances of gut microbiota composition (e.g., regulating *Alternaria* and *Pichia* abundances), initiating a signaling cascade to activates key metabolites (14,15-DiHETrE, 5-HIAA, and prostaglandin C2) to suppress oxidative stress damage. Modulates *Fas*/*Tnfsf10*-mediated apoptosis pathways in intestinal, liver, and brain tissues. This process forms a synergistic regulatory network of the fungi-metabolite-genes, thereby exerting protective effects against acute oxidative stress.

To elucidate the structure–activity relationship of SBE in combating oxidative stress, LC–MS/MS was used for qualitative analysis of its active ingredients (Supplementary Fig.2 and Supplementary Table 10). Network pharmacology combined with molecular docking was then employed to identify the targets of these components. The results demonstrated that trigonelline, mesaconic acid, and salicylic acid may be as key active constituents acting on Fas and Tnfsf10 in the apoptotic signaling pathway**.** Notably, target proteins displayed diverse binding sites for the key compounds of SBE (Fig. 5F–G). Such as hydrogen-bonding interactions between trigonelline and Fas at LEU-165, GLN-446, and ARG-384 sites; hydrogen-bonding interactions between mesaconic acid and Fas at ARG-708 and ARG-728 sites; and hydrogen-bonding interactions between salicylic acid and Fas at the ALA-727, ARG-728, ARG-708, and SER-709 sites. In addition, hydrogen-bonding interactions between trigonelline and Tnfsf10 at the ALA-140, LEU-141 sites, hydrogen-bonding interactions between mesaconic acid and Tnfsf10 at the GLU-198, SER-242, ARG-212 sites, and hydrogen-bonding interactions between salicylic acid and Tnfsf10 at the ARG-135 and LEU-278 sites. Moreover, the binding energy between Tnsfs10 and molecules was lower than Fas (Supplementary Table 11).

## Discussion

Ethanol exposure induces oxidative stress through a synergistic interplay multiple mechanisms. For example, it reduces GSH levels in both mitochondria and cytoplasm, impairs mitochondrial ability to scavenge ROS, promotes the formation of membrane lipid peroxidation products, and inhibits antioxidant enzymes such as GSH-Px. The cascade weakens the cellular antioxidant defenses, resulting in organelle membrane damage and compromised enzyme activity [41]. In acute ethanol exposure models, systemic oxidative stress responses are rapidly triggered in mice, affecting the tissues such as brain [49], liver [50, 51], intestine [52], and so on [41, 43]. Consistently, ROS activation is significantly observed in the stomach, small intestine, cecum, and colon of mice after alcohol gavage (Supplementary material Result 1).

The antioxidant properties of SBE not only suppress ROS production (Supplementary material Result 1) and lipid peroxidation but also decrease PC levels while enhancing the activities of specific antioxidant enzymes (e.g., GSH-Px, CAT, SOD) and the level of GSH in serum, as well as in the intestine, liver, and brain tissues (Fig. 1). However, redox homeostasis represents a dynamically maintained process regulated by integrated prevention, interception, and repair mechanisms. Regulatory components, including molecular thiol-driven master switches, nonradical species, and enzymatic defense systems, collectively orchestrate the fine-tuning of physiological redox signaling to sustain systemic homeostasis [53]. Accordingly, not all assayed oxidative stress biomarkers in this study exhibited significant alterations, a finding that reflects the complex regulatory networks underlying redox balance.

ROS can directly interact with the tight junction complex, disrupting the cytoskeleton, altering paracellular permeability, and destabilizing tight junctions, thereby compromising barrier function [54–56]. Colonic tissues are particularly vulnerable to oxidative stress-induced inflammation, as supported by previous studies [57–59]. Histopathological analysis revealed that SBE effectively mitigated ethanol-induced damage, including reducing the infiltration of intestinal inflammatory cells and hepatic parenchymal cell steatosis, and alleviating hippocampal neuronal injury (Fig. 1B–D(h)). Additionally, SBE reconstituted intestinal barrier function by up-regulating the expression of key tight junction proteins, Claudin-1 and E-cadherin (Fig. 1B(i)).

Taken together, SBE exerts antioxidant properties by inhibiting ROS generation and lipid peroxidation, enhancing the activities of antioxidant enzymes, maintaining GSH levels, and repairing tissue barrier function through multi-target synergistic modulation of ethanol-induced oxidative stress damage. These findings align with established mechanisms, where natural products typically alleviate oxidative stress via free radical scavenging and activation of antioxidant enzyme systems [36, 37].

The gut microbiota is predominantly composed of bacteria, with over 99% of its members belonging to this domain. Previous studies have predominantly focused on the relationship between oxidative stress and gut bacteria [40, 60, 61]. However, our study revealed that SBE significantly modulated the composition of intestinal fungi. Notably, when our research team initially demonstrated that SBE restored the intestinal microbiota balance in an ethanol-induced oxidative stress mouse model, the pivotal role of fungal communities in this process was an unexpected discovery. A particularly striking finding was the strength of the association between fungi and oxidative stress. Our analysis showed that 74 fungal species were significantly correlated (*p* < 0.05) with oxidative stress biomarkers, surpassing the 49 bacterial species identified with such correlations (Fig. 2). This observation aligns with emerging evidence from other studies [2, 62–64], which suggest that gut fungi represent a crucial yet understudied component of the microbiota, playing important roles in nutrient metabolism and gut health in both humans and animals. Collectively, these results indicate that the long-neglected fungal microbiome may serve as a key regulatory element in oxidative stress responses.

Multimodal correlation analysis revealed that *Alternaria* abundance positively correlated with serum PC levels but negatively with serum GSH levels (*p* < 0.05). However, previous research has shown that *Alternaria*-produced mycotoxin altertoxin II (ATX II) can activate the Nrf2-ARE pathway in a concentration-dependent manner, inducing Nrf2 nuclear translocation and increasing the transcription of γ-glutamylcysteine ligase (γGCL), resulting in biphasic changes in intracellular GSH levels (initially decreasing followed by an increase) [65]. Streptavidin also functions as a potent Nrf2 activator in PC12 cells, exhibiting significant neuroprotective effects against oxidative damage [66]. Our previous studies have demonstrated that SBE activates the Nrf2-Keap1-ARE signaling pathway [39]. The observed changes in *Alternaria* abundance following SBE intervention likely reflect the activation of the Nrf2-ARE pathway, triggering an adaptive antioxidant response. Nrf2 activation induces the transcription of antioxidant enzymes such as γGCL, enhancing GSH synthesis capacity and contributing to increased GSH levels in various tissues (Fig. 1).

These fungal abundance changes were significantly associated with the expression of metabolites in the serotonergic synaptic pathway (14,15-DiHETrE, 5-HIAA, prostaglandin C2) and genes in the apoptotic signaling pathway (*Fas* and *Tnfsf10*). The Spearman correlation analysis further demonstrated that 14,15-DiHETrE, 5-HIAA, and prostaglandin C2 were significantly associated with the expression of *Fas* and *Tnfsf10* in the apoptotic pathway, suggesting a potential regulatory network (Fig. 5A). However, these correlations highlight the interplay among fungi, metabolites, and genes, future studies are warranted to elucidate the underlying mechanisms through in-depth functional validation.

The expression level of *Fas* was positively correlated with that of 14,15-DiHETrE while *Alternaria* negatively correlated with both (*p* < 0.05). *Fas*, a member of the TNF receptor superfamily, mediates apoptosis and contributes to T cell activation, tumor progression, and metabolic dysregulation [67, 68]. Eliminating *Fas* in liver cells mitigates hepatic steatosis, consistent with our findings, indicating that SBE can improve liver tissue injury by reducing the expression of *Fas *[69]*. Fas* signaling activation exacerbates oxidative stress through dual mechanisms: activating NADPH oxidase to promote mitochondrial ROS generation, disrupting mitochondrial membrane potential, releasing cytochrome c, and exacerbating oxidative damage; and inhibiting antioxidant enzyme activities (e.g., GSH-Px and SOD activities), which diminishes cellular antioxidant capacity [32]. Conversely, the inhibition of *Fas* signaling alleviates oxidative stress damage. Our findings thus demonstrate that SBE suppresses *Fas* signaling, thereby reducing oxidative stress.

Oxidative stress also plays an important role in the process of virus-induced liver cancer (HCC), causing a significant increase in the level of 14,15-DiHETrE that was found to be associated with the levels of tumor marker α-fetoprotein (AFP) [44]. Our results also indicate that oxidative stress increased the level of 14,15-DiHETrE, while SBE decreased it (Fig. 5B). 14,15-DiHETrE (14,15-dihydroxyeicosatetraenoic acid) is a cytochrome P450 (CYP)-derived arachidonic acid metabolite that induces the binding of PPARs to peroxisome proliferator response elements (PPREs) in the promoter regions of target genes [70, 71]. Previous studies have demonstrated that leucine activates PPARs, thereby promoting mitochondrial biogenesis and oxidative metabolism. This activation also enhances the expression of *SREBP-1c* and *FAS*, ultimately increasing fatty acid synthesis and lipid storage [72]. Our findings suggest that 14,15-DiHETrE may exert similar effects (Fig. 5E). Our results indicate that SBE may activate antioxidant pathways by regulating *Alternaria* abundance, thereby inhibiting *Fas* expression and 14,15-DiHETrE secretion, and establishing a more robust antioxidant defense system. Based on these findings, we propose that SBE may activate the Nrf2-Keap1-ARE antioxidant pathway by modulating *Alternaria* abundance. Additionally, SBE could potentially reduce the expression of *Fas* by inhibiting the production of 14,15-DiHETrE.

Conversely, *Pichia* abundance showed positive associations with serum and brain 8-isoprostane and PC levels, while negatively correlating with liver SOD, CAT, and GSH levels (*p* < 0.05). SBE treatment reduces the abundance of *Pichia*, enhances antioxidant enzyme activity, and alleviates oxidative stress. Unlike *Pichia, Bifidobacterium,* and lactic acid bacteria (LAB) promote the synthesis of antioxidant enzymes (e.g., GSH-Px, CAT, and SOD), thereby enhancing the host’s capacity to scavenge ROS [73]. Regarding *Pichia*, previous studies have reported its potential pro-inflammatory effects [74, 75], which are inseparable from oxidative stress. In our study, SBE intervention reduced the abundance of *Pichia*. Moreover, the abundance of *Pichia* was positively correlated with 5-HIAA, prostaglandin C2, and *Tnfsf10* (Fig. 5A). *TNFSF10*, as an oxidative stress inducer, activates mitochondrial ROS generation, triggers N-terminal arginylation of HSPA5 and oxidation of PARK7, and thereby initiates the autophagic protein degradation pathway. *TNFSF10* impairs the binding of KEAP1 to SQSTM1, which affects the release of the Nrf2 transcription factor and may indirectly inhibit antioxidant gene expression [76]. Furthermore, *TNFSF10* is positively correlated with the expression of oxidative stress markers (such as H₂O₂) and p53 [76]. Under oxidative stress conditions (e.g., H₂O₂ treatment), *TNFSF10* induces apoptosis, consuming antioxidant enzymes (such as SOD) and intensifying mitochondrial ROS generation [77]. Our results demonstrate that following treatment with SBE, the expression of *Tnfsf10* was down-regulated, thereby modulating the levels of oxidative stress markers. The conversion of 5-hydroxytryptamine (5-HT) to 5-HIAA by mitochondrial monoamine oxidase A (MAO-A) generates H₂O₂ and ROS, which leads to oxidative stress, and the 5-HIAA/5-HT ratio thus serves as a marker of oxidative stress and inflammation [78–81]. In the oxidative stress-induced inflammatory response, the level of prostaglandin C2 was increased [82], while *Tnfsf10* levels correlate with ethanol relapse risk [83–85]. Consistent with these findings, ethanol increased *Tnfsf10* expression in intestinal, liver, and brain tissues, which was reversed by SBE (Fig. 4C). Based on the foregoing evidence, we speculate that SBE inhibits ROS production induced by high *Tnfsf10* expression by downregulating *Pichia* abundance. Furthermore, SBE activates the Nrf2 antioxidant pathway, thereby reducing H₂O₂ generation during 5-HIAA conversion and decreasing levels of prostaglandin C2, an oxidative stress-induced inflammatory mediator. These mechanisms collectively disrupt the oxidative stress–inflammatory response vicious cycle.

This study, for the first time, employed multi-omics integration to elucidate the mechanism by which SBE alleviates oxidative stress through the regulation of a “fungal-metabolic-gene” interaction network (Fig. 5A–E).

To further explore the mechanism by which SBE mitigates oxidative stress, we investigated the structure–activity relationship of its active components (Fig. 5F). Trigonelline, an alkaloid, has been shown to possess antioxidant properties, enhance glucose tolerance, and exhibit neuroprotective effects against various neurological disorders [86, 87]. Mesaconic acid, as an intermediate in the citric acid cycle, plays a vital role in aging and immune regulation [88]. Salicylic acid, well-known for its anti-inflammatory and keratin-conditioning properties, is widely used in pharmaceuticals and cosmetics [89]. The distinct structures of these components likely contribute to their differential efficacies: trigonelline contains a pyridine ring; mesaconic acid is a dicarboxylic acid with two carboxyl groups and a double bond; and salicylic acid is an o-hydroxybenzoic acid featuring one carboxyl and phenolic hydroxyl group.

Molecular docking results revealed differential binding patterns of trigonelline, mesaconic acid, and salicylic acid to the Tnfsf10 and Fas targets (Fig. 5F). Mesaconic acid and salicylic acid, both bearing carboxyl groups, interact with the Fas target at common binding sites ARG-728 and ARG-708. Although trigonelline lacks a free carboxyl group—it is an imino betaine derived from carboxyl group deprotonation—it binds to the Fas target at ARG-384. Our findings indicate that the carboxyl group enhances the compound’s affinity for hydroxyl-containing residues, independent of the number of carboxyl groups present (Supplementary Table 11 and Supplementary Fig.2). Carboxyl groups can enhance the chelating ability of metals and reduce the generation of free radicals by forming intramolecular hydrogen bonds [90, 91]. This suggests that the carboxyl group is a key functional moiety for modulating the Fas target, with ARG-728 and ARG-708 serving as critical binding sites. For the Tnfsf10 target, salicylic acid exhibits the highest affinity, likely attributed to its phenolic hydroxyl group. The incorporation of phenolic hydroxyl groups significantly enhances the antioxidant capacity of phenolic acids, which is intricately associated with thermodynamic parameters governing radical-scavenging mechanisms [92, 93]. The presence of ARG and LEU residues at the binding interface further supports this high-affinity interaction, indicating that the phenolic hydroxyl group is a crucial determinant for Tnfsf10 modulation, and ARG and LEU are key binding sites.

Collectively, these results suggest that SBE may exert its antioxidant effects through a multi-target mechanism, with trigonelline, mesaconic acid, and salicylic acid binding to Tnfsf10 and Fas in the apoptotic signaling pathway (Fig. 5G). Future studies will focus on experimentally validating these binding interactions and elucidating the downstream signaling cascades activated by these component-target binging, which will address the current limitations of this work.

Considering that SBE exerts antioxidant stress effects through a multi-component and multi-target mechanism, its potential applications encompass diverse fields. For instance, SBE has been shown to mitigate oxidative damage in intestinal, liver, and brain tissues induced by acute alcohol exposure—pathologies intimately linked to hangover remedies. Given the close association between hangover relief and oxidative stress modulation (such as increasing the activity of SOD and GSH-Px, elevating GSH content, and reducing MDA content [94]), SBE warrants consideration as a functional ingredient for developing hangover-relieving and liver-protective health products. Oxidative stress interacts with intestinal flora imbalance, mediating the occurrence of neurodegenerative diseases [9]. SBE-mediated regulation of apoptotic signaling pathways and other key molecular cascades positions it as a promising candidate for developing novel therapeutic agents or nutraceutical supplements in the management of neurodegenerative disorders [95–97]. By simultaneously targeting multiple pathophysiological pathways, SBE has the potential to enhance the efficacy of existing therapies and improve patient outcomes.

## Conclusions

Here, ethanol gavage in mice leads to a decrease in GSH levels and ROS scavenging capacity, accompanied by increased production of lipid peroxidation products (e.g., MDA, isoprostanes) and inhibition of antioxidant enzyme activities, including SOD, CAT, and GSH-Px. In contrast, administration of SBE reduces the production of MDA, 8-isoprostane, and PC, while increasing the activity of SOD, CAT, and GSH-Px. This synergistic modulation elevates intracellular GSH levels and reconstitutes the antioxidant defense system, ultimately culminating in a marked decrease in ROS levels and a systemic enhancement of the mice’s antioxidant capacity. Spearman correlation analysis identified a functional triad: *Alternaria* abundance negatively correlated with pro-oxidative metabolites (14,15-DiHETrE, prostaglandin C2) and apoptotic genes (*Fas*, *Tnfsf10*), whereas *Pichia* showed a positive association with these stress biomarkers. Network analysis mapped this microbial-metabolite-gene axis to fungal species regulation, serotonergic synapse and apoptotic signaling pathways, underscoring a multi-pathway and multi-target regulatory mechanism. Molecular docking further showed that the active components of SBE (trigonelline, mesaconic acid, salicylic acid) exhibited high binding affinity for Fas and Tnfsf10 targets, thereby mechanistically linking structural interactions to pathway modulation. Collectively, these findings establish SBE’s antioxidant efficacy as a microbiota-mediated, multi-target process. Notwithstanding our extensive investigations, certain mechanistic details (e.g., specific microbial metabolites’ roles) remain to be elucidated. This work provides a theoretical framework for understanding SBE’s antioxidant mechanism, paving the way for subsequent mechanistic investigations and translational applications.

## Supplementary Information

 Supplementary material 1.

## Data Availability

The research paper contains the data that underpins the conclusions drawn in this study. The NCBI Sequence Read Archive (SRA) hosts the gut bacterial and fungal datasets that support the findings of this study under the BioProjects PRJNA1105339 and PRJNA1105342, respectively. The metabolomics data in the paper have been deposited in the OMIX of China National Center for Bioinformation (https://ngdc.cncb.ac.cn/omix: accession no.OMIX011440) under the BioProject PRJCA044634. The NCBI Sequence Read Archive (SRA) hosts transcriptomic datasets produced in this work under BioProject PRJNA1105673. The prediction of the protein–protein interactions was performed using the STRING database (http://string-db.org/). The prediction of the targets was performed using BATMAN-TCM (http://bionet.ncpsb.org.cn/batman-tcm/#/home), Swiss Target Prediction (http://www.swisstargetprediction.ch) and ChEMBL (https://www.ebi.ac.uk/chembl/). Additional source data are available by contacting the corresponding author.
