# Supplementary material for: Regulation on microbial composition,serotonergic synapse, and apoptotic signaling pathway by extracts fromSonchus brachyotus DC. (SBE) to improve ethanol-induced acute oxidative stress in mice
Source: Microbiome. 2025 Oct 29;13:221. doi: 10.1186/s40168-025-02221-8 (PMC12573829; doi:10.1186/s40168-025-02221-8)
Supplement: Supplementary file 2 — Supplementary material 1. [file 40168_2025_2221_MOESM1_ESM.docx]

**Supplementary materials**

**Result 1. Establishment of an ethanol-induced acute oxidative stress model in mice**

To determine the results of edible alcohol-induced systemic acute oxidative stress in mice, we assayed the levels of ROS in vivo (Supplementary Fig.1). The results showed that the sites with obvious ROS production were the cecum and parts of the stomach and small intestine, and the ROS level was increased in the EtOH group, while the ROS level decreased and returned to the CON group level in all T groups. The results indicated that SBE could effectively reduce the alcohol-induced elevated ROS levels and keep the balance of ROS levels in mice.

**
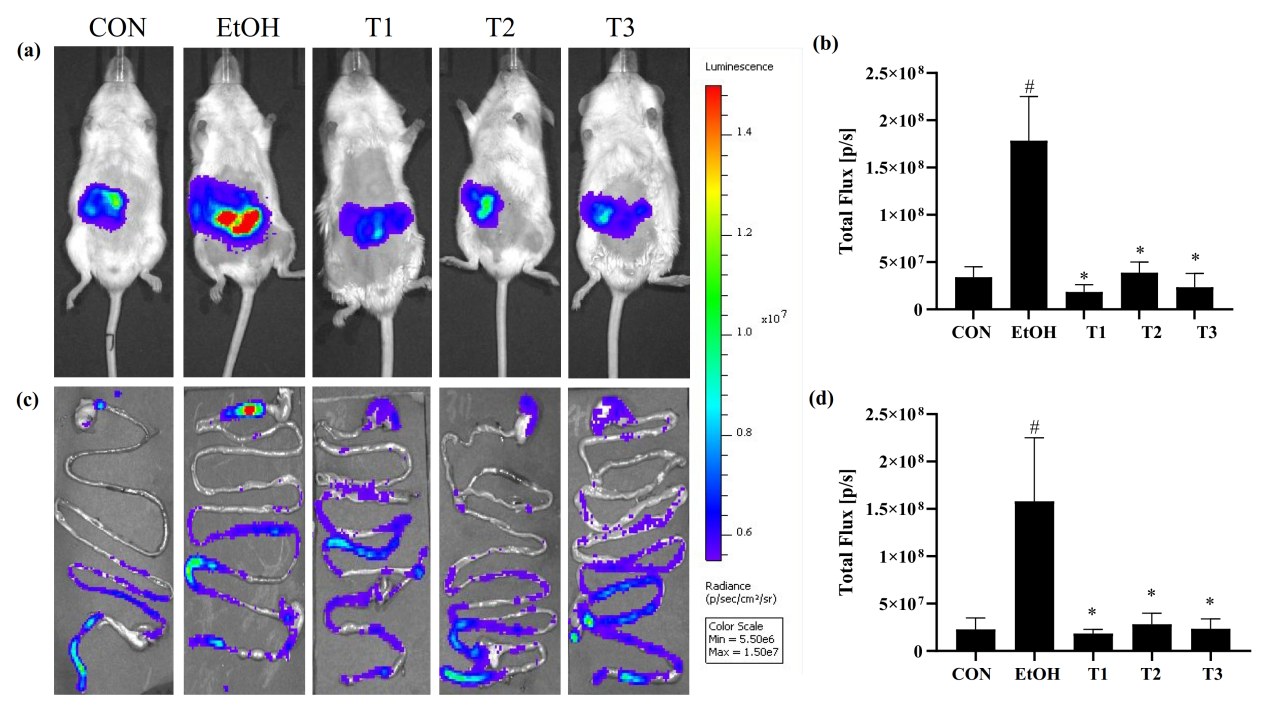
**

**Supplementary Fig.1** The biological imaging system analyzed the effect of alcohol on ROS levels in mice. The colors represent the production of ROS fluorescence, with red indicating high ROS levels and blue indicating low ROS levels. The thermal image of the level of ROS in the mice was captured by an in vivo imaging system (a). The statistical bar graph of the fluorescence value of ROS in mice (b). The thermal image of the variation of the level of ROS in the digestive tract of the mice (c). The statistical bar graph of the fluorescence value of ROS in the digestive tract of the mice (d).

**Result 2. Effect of SBE on growth performance in mice**

We investigated the effect of SBE on the growth performance of mice and noticed that a dose-dependent relationship was observed in the total body weight gain of mice in the T groups when compared with the CON and EtOH groups, but the difference was not significant (Supplementary Table 1). Compared with the CON and EtOH groups, the spleen weight index was increased in the T groups, which was significantly increased in the T1 group (*p* < 0.05). The spleen, a peripheral immune organ, whose weight index can reflect the immune function of the organism, which suggests that SBE increases the immune ability of mice. There was no significant change in the liver weight index between the different treatment groups, suggesting that SBE does not burden the liver in mice.

Supplementary Table 1 Effects of SBE on the growth performance of mice

| Items | CON | EtOH | T1 | T2 | T3 | *P* |
| --- | --- | --- | --- | --- | --- | --- |
| Initial body weight （g/each） | 27.47 ± 0.55 | 27.18 ± 0.51 | 27.37 ± 0.47 | 27.28 ± 0.49 | 27.28 ± 0.69 | 0.5797 |
| Final body weight （g/each） | 39.24 ± 0.71 | 39.28 ± 0.86 | 39.71 ± 0.56 | 39.84 ± 0.73 | 41.62 ± 1.04 | 0.2170 |
| Body weight gain （g/each） | 11.77 ± 0.51 | 12.1 ± 0.43 | 12.34 ± 0.35 | 12.56 ± 0.46 | 13.28 ± 0.68 | 0.2793 |
| Feed intake （g/d） | 5.67 ± 0.07 | 5.72 ± 0.09 | 5.75 ± 0.09 | 5.55 ± 0.05 | 5.99 ± 0.08 | 0.0125 |
| Feed conversion ratio | 5.24% | 5.34% | 5.94% | 6.24% | 6.10% |  |
| Spleen weight （g/each） | 0.08 ± 0.01 | 0.08 ± 0.01 | 0.11 ± 0.01 | 0.10 ± 0.01 | 0.10 ± 0.01 | 0.0172 |
| Liver weight （g/each） | 1.58 ± 0.06 | 1.67 ± 0.04 | 1.68 ± 0.05 | 1.62 ± 0.03 | 1.72 ± 0.06 | 0.0994 |

**Result 3. Effect of SBE on biochemical indexes in mice**

From the study of the effect of SBE on serum biochemical indexes of mice (Supplementary Table 2), T-CHO levels in serum of mice in all T groups decreased significantly (*p* < 0.05) compared with the EtOH group, and the T1 group exhibited the lowest T-CHO levels. The TG levels in all T groups decreased significantly (*p* < 0.05), which showed a dose-dependent relationship with the dosage of SBE. There was no significant difference in the levels of ADH, ALT, AST, and LDL-C among all groups when compared. The results indicated that SBE effectively reduced serum T-CHO and TG levels.

Supplementary Table 2 Effects of SBE on serum biochemical indexes in mice

| Items | | CON | | EtOH | | T1 | | T2 | | T3 | *P* value | |
| --- | --- | --- | --- | --- | --- | --- | --- | --- | --- | --- | --- | --- |
| T-CHO（mmol/L） | | 4.29 ± 0.15 | | 4.66 ± 0.40 | | 3.06 ± 0.21 | | 3.84 ± 0.27* | | 3.84 ± 0.27* | 0.003 | |
| TG（mmol/L） | | 1.58 ± 0.18 | | 1.99 ± 0.21 | | 1.44 ± 0.16 | | 1.34 ± 0.15* | | 1.31 ± 0.11* | 0.013 | |
| ADH（U/mL） | | 17.14 ± 2.05 | | 10.75 ± 2.54 | | 11.91 ± 1.29 | | 11.83 ± 1.39 | | 13.63 ± 2.64 | 0.103 | |
| ALT(U/L) | | 45.27 ± 1.06 | | 51.10 ± 5.6 | | 44.86 ± 0.73 | | 45.65 ± 1.12 | | 48.63 ± 2.64 | 0.571 | |
| AST(U/L) | 28.13 ± 1.35 | | 38.38 ± 4.48 | | 35.2 ± 2.45 | | 35.58 ± 2.76 | | 34.23 ± 2.66 | | 0.064 |  |
| LDL-C (mmol/L) | 0.20 ± 0.03 | | 0.44 ± 0.04 | | 0.49 ± 0.03 | | 0.54 ± 0.04 | | 0.37 ± 0.05 | | 0.746 |  |

Note: * *p* <0.05, ** *p* <0.01

The study of the effect of SBE on the biochemical indexes of liver tissue revealed that it reduced the level of T-CHO in liver tissue in all T groups compared with the EtOH group, and the level of T-CHO in the T1 group was significantly reduced (*p* < 0.05), which was in accordance with the results of serum. Meanwhile, the level of TG in liver tissue in all T groups was reduced compared with the EtOH group, and the level of TG in the T2 group was significantly reduced (*p* < 0.05). The regulation effect of SBE on ADH, ALT, AST, and LDL-C was consistent with that of serum, which suggested SBE should positively regulated lipid metabolism in mice.

Supplementary Table 3 Effects of SBE on liver biochemical indexes in mice

| Group | CON | EtOH | T1 | T2 | T3 | | *P* value | |
| --- | --- | --- | --- | --- | --- | --- | --- | --- |
| T-CHO（mmol/L） | 0.15 ± 0.02 | 0.17 ± 0.02 | 0.04 ± 0.01** | 0.11 ± 0.01 | 0.11 ± 0.02 | 0.002 | |  |
| TG（mmol/L） | 0.08 ± 0.02 | 0.16 ± 0.02 | 0.10 ± 0.01 | 0.05 ± 0.02* | 0.09 ± 0.01 | 0.028 | |  |
| ADH（U/mL） | 1.17 ± 0.54 | 1.47 ± 0.22 | 1.61 ± 0.22 | 1.09 ± 0.18 | 0.89 ± 0.24 | 0.133 | |  |
| ALT(U/L) | 52.16 ± 9.77 | 43.63 ± 4.05 | 42.95 ± 5.33 | 42.79 ± 3.13 | 36.87 ± 4.73 | 0.986 | |  |
| AST(U/L) | 122.26 ± 24.09 | 155.85 ± 28.28 | 142.12 ± 10.44 | 135.52 ± 18.36 | 135.3 ± 10.89 | 0.965 | |  |
| LDL-C (mmol/L) | 0.03 ± 0.00 | 0.13 ± 0.04 | 0.04 ± 0.01 | 0.05 ± 0.01 | 0.04 ± 0.01 | 0.062 | |  |

Note: **p* <0.05, ** *p* <0.01

Supplementary Table 4 Raw reads of bacteria

| Sample | Kingdom | Phylum | Class | Order | Family | Genus | Species |
| --- | --- | --- | --- | --- | --- | --- | --- |
| C-1 | 9824 | 9824 | 9824 | 9808 | 9576 | 8238 | 708 |
| C-2 | 11194 | 11194 | 11194 | 11175 | 11051 | 8770 | 1252 |
| C-3 | 11906 | 11906 | 11906 | 11884 | 11590 | 8272 | 896 |
| C-4 | 10098 | 10098 | 10098 | 10090 | 10017 | 8747 | 2199 |
| C-5 | 11830 | 11830 | 11830 | 11829 | 11315 | 8377 | 4509 |
| C-6 | 11881 | 11881 | 11881 | 11848 | 11030 | 7414 | 1479 |
| M-1 | 10295 | 10295 | 10295 | 10295 | 10179 | 7648 | 6659 |
| M-2 | 10810 | 10810 | 10810 | 10798 | 10335 | 7187 | 3512 |
| M-3 | 8485 | 8485 | 8485 | 8484 | 8038 | 6124 | 4650 |
| M-4 | 11963 | 11963 | 11963 | 11963 | 11786 | 10213 | 2070 |
| M-5 | 11094 | 11094 | 11094 | 11094 | 11091 | 11089 | 10845 |
| M-6 | 11072 | 11072 | 11072 | 11059 | 10322 | 6391 | 716 |
| T1-1 | 9413 | 9413 | 9413 | 9413 | 9079 | 5480 | 1712 |
| T1-2 | 10839 | 10839 | 10839 | 10830 | 10154 | 6918 | 3811 |
| T1-3 | 11780 | 11780 | 11780 | 11755 | 9775 | 6929 | 2924 |
| T1-4 | 11336 | 11336 | 11336 | 11324 | 10936 | 6303 | 4093 |
| T1-5 | 11277 | 11277 | 11277 | 11276 | 11144 | 8720 | 2570 |
| T1-6 | 10276 | 10276 | 10276 | 10273 | 10221 | 8631 | 7316 |
| T2-1 | 9046 | 9046 | 9046 | 9039 | 8603 | 4717 | 1657 |
| T2-2 | 9326 | 9326 | 9326 | 9306 | 8951 | 5558 | 1169 |
| T2-3 | 11564 | 11564 | 11564 | 11559 | 11313 | 6504 | 3037 |
| T2-4 | 9682 | 9682 | 9682 | 9682 | 9649 | 4885 | 1303 |
| T2-5 | 11541 | 11541 | 11541 | 11540 | 10724 | 8248 | 5935 |
| T2-6 | 8014 | 8014 | 8014 | 8004 | 7647 | 4870 | 1556 |
| T3-1 | 9052 | 9052 | 9052 | 9051 | 8966 | 7495 | 1582 |
| T3-2 | 9143 | 9143 | 9143 | 9139 | 8904 | 6431 | 4948 |
| T3-3 | 10849 | 10849 | 10849 | 10847 | 10670 | 6596 | 2138 |
| T3-4 | 11411 | 11411 | 11411 | 11411 | 11036 | 5822 | 2565 |
| T3-5 | 9751 | 9751 | 9751 | 9740 | 9151 | 5422 | 1846 |
| T3-6 | 9543 | 9543 | 9543 | 9508 | 9235 | 6688 | 647 |

* The above table shows the Reads statistics of each level of the bacterial sample, where the values represent the total number of Reads covered by this level of the sample

Supplementary Table 5 Raw reads of fungi

| Sample | Kingdom | Phylum | Class | Order | Family | Genus | Species |
| --- | --- | --- | --- | --- | --- | --- | --- |
| C-1 | 10448 | 10448 | 10448 | 10448 | 10448 | 10448 | 10448 |
| C-2 | 11896 | 11896 | 11896 | 11896 | 11896 | 11896 | 11896 |
| C-3 | 11299 | 11299 | 11299 | 11299 | 11299 | 11299 | 11299 |
| C-4 | 11716 | 11716 | 11716 | 11716 | 11716 | 11716 | 11716 |
| C-5 | 11681 | 11681 | 11681 | 11681 | 11681 | 11681 | 11681 |
| C-6 | 11743 | 11743 | 11743 | 11743 | 11743 | 11743 | 11743 |
| M-1 | 11770 | 11770 | 11770 | 11770 | 11770 | 11770 | 11770 |
| M-2 | 11792 | 11792 | 11792 | 11792 | 11792 | 11792 | 11792 |
| M-3 | 11581 | 11581 | 11581 | 11581 | 11581 | 11581 | 11581 |
| M-4 | 11632 | 11632 | 11632 | 11632 | 11632 | 11632 | 11632 |
| M-5 | 11884 | 11884 | 11884 | 11884 | 11884 | 11884 | 11884 |
| M-6 | 11807 | 11807 | 11807 | 11807 | 11807 | 11807 | 11807 |
| T1-1 | 12336 | 12336 | 12336 | 12336 | 12336 | 12336 | 12336 |
| T1-2 | 11693 | 11693 | 11693 | 11693 | 11693 | 11693 | 11693 |
| T1-3 | 11987 | 11987 | 11987 | 11987 | 11987 | 11987 | 11987 |
| T1-4 | 12568 | 12568 | 12568 | 12568 | 12568 | 12568 | 12568 |
| T1-5 | 10197 | 10197 | 10197 | 10197 | 10197 | 10197 | 10197 |
| T1-6 | 11740 | 11740 | 11740 | 11740 | 11740 | 11740 | 11740 |
| T2-1 | 12197 | 12197 | 12197 | 12197 | 12197 | 12197 | 12197 |
| T2-2 | 12349 | 12349 | 12349 | 12349 | 12349 | 12349 | 12349 |
| T2-3 | 12360 | 12360 | 12360 | 12360 | 12360 | 12360 | 12360 |
| T2-4 | 12491 | 12491 | 12491 | 12491 | 12491 | 12491 | 12491 |
| T2-5 | 12658 | 12658 | 12658 | 12658 | 12658 | 12658 | 12658 |
| T2-6 | 12464 | 12464 | 12464 | 12464 | 12464 | 12464 | 12464 |
| T3-1 | 10250 | 10250 | 10250 | 10250 | 10250 | 10250 | 10250 |
| T3-2 | 11868 | 11868 | 11868 | 11868 | 11868 | 11868 | 11868 |
| T3-3 | 12288 | 12288 | 12288 | 12288 | 12288 | 12288 | 12288 |
| T3-4 | 12121 | 12121 | 12121 | 12121 | 12121 | 12121 | 12121 |
| T3-5 | 12163 | 12163 | 12163 | 12163 | 12163 | 12163 | 12163 |
| T3-6 | 12364 | 12364 | 12364 | 12364 | 12364 | 12364 | 12364 |

* The above table shows the Reads statistics of each level of the fungal sample, where the values represent the total number of Reads covered by this level of the sample

Supplementary Table 6 Statistical table of DEMs for differential groups

| **Group** | **DEMs_total** | **DEMs_up** | **DEMs_down** |
| --- | --- | --- | --- |
| CON_vs_EtOH | 387 | 314 | 73 |
| EtOH_vs_T1 | 298 | 186 | 112 |
| EtOH_vs_T2 | 137 | 57 | 80 |
| EtOH_vs_T3 | 163 | 41 | 122 |

Supplementary Table 7 The DEMS of Serotonergic synapse pathway

| Metabolites | FC | VIP | Relationship | Group |
| --- | --- | --- | --- | --- |
| 5-Hydroxyindole-3-acetic acid | 2.20 ^**^ | 1.43 | up | CON vs EtOH |
| 14,15-DiHETrE | 2.65 ^**^ | 1.34 | up |  |
| Prostaglandin C2 | 2.66 ^**^ | 1.43 | up |  |
| 5,6-DHET | 9.78 ^**^ | 1.43 | up |  |
| Metabolites | FC | VIP | Relationship | Group |
| 5-Hydroxyindole-3-acetic acid | 1.20 | 1.30 | unchanged | EtOH vs T1 |
| 14,15-DiHETrE | 3.31 ** | 1.42 | down |  |
| Prostaglandin C2 | 2.42** | 1.35 | down |  |
| 5,6-DHET | 8.60 ** | 1.47 | down |  |
| 5-Hydroxyindole-3-acetic acid | 1.00 | 0.03 | unchanged | EtOH vs T2 |
| 14,15-DiHETrE | 1.84 | 1.46 | unchanged |  |
| Prostaglandin C2 | 2.91** | 1.59 | down |  |
| 5,6-DHET | 5.06 ** | 1.61 | down |  |
| 5-Hydroxyindole-3-acetic acid | 1.46 | 0.53 | unchanged | EtOH vs T3 |
| 14,15-DiHETrE | 2.09* | 1.71 | down |  |
| Prostaglandin C2 | 3.34 | 1.59 | unchanged |  |
| 5,6-DHET | 6.41 | 1.05 | unchanged |  |

**p*<0.05; ** p<0.01

Supplementary Table 8 Summary table of the number of differential gene expressions between groups

| Screening condition | Group | DEGs | Up-DEGs | Down-DEGs |
| --- | --- | --- | --- | --- |
| DESeq2_EBSeq  FDR=0.01 FC=1.5 | CON_vs_EtOH | 8 | 3 | 5 |
|  | EtOH_vs_T1 | 0 | 0 | 0 |
|  | EtOH_vs_T2 | 7 | 1 | 6 |
|  | EtOH_vs_T3 | 9 | 0 | 9 |

Supplementary Table 9 Bacteria-metabolite-gene interaction results

| **ID1** | **ID2** | **coefficient** | ***p*** | **ID1 type** | **ID2 type** |
| --- | --- | --- | --- | --- | --- |
| 14,15-DiHETrE | *Bacteroides* | 0.83 | 0.04 | metabolite | genus |
| 14,15-DiHETrE | gene-Rab19 | 0.89 | 0.02 | metabolite | gene |
| 14,15-DiHETrE | gene-*Fas* | 0.83 | 0.04 | metabolite | gene |
| 14,15-DiHETrE | *Aspergillus* | -0.83 | 0.04 | metabolite | genus |
| 14,15-DiHETrE | *Alternaria* | -0.89 | 0.02 | metabolite | genus |
| 5,6-DHET | *uncultured_bacterium_f_Muribaculaceae* | 0.94 | 0.00 | metabolite | genus |
| 5,6-DHET | *Bacteroides* | 1.00 | 0.00 | metabolite | genus |
| 5,6-DHET | gene-*Rpl21* | -0.83 | 0.04 | metabolite | gene |
| 5,6-DHET | gene-Rab19 | 0.89 | 0.02 | metabolite | gene |
| 5-Hydroxyindole-3-acetic acid | *uncultured_bacterium_f_Muribaculaceae* | 0.83 | 0.04 | metabolite | genus |
| 5-Hydroxyindole-3-acetic acid | *Erysipelatoclostridium* | -0.83 | 0.04 | metabolite | genus |
| 5-Hydroxyindole-3-acetic acid | gene-*C2cd4b* | -0.94 | 0.00 | metabolite | gene |
| 5-Hydroxyindole-3-acetic acid | gene-*Rpl21* | -0.94 | 0.00 | metabolite | gene |
| 5-Hydroxyindole-3-acetic acid | gene-*Tnfsf10* | 0.83 | 0.04 | metabolite | gene |
| 5-Hydroxyindole-3-acetic acid | gene-*Rasef* | -0.83 | 0.04 | metabolite | gene |
| 5-Hydroxyindole-3-acetic acid | *Pichia* | 1.00 | 0.00 | metabolite | genus |
| *Alistipes* | gene-*Etv4* | 0.94 | 0.00 | genus | gene |
| *Alistipes* | gene-*Tnfsf10* | -0.89 | 0.02 | genus | gene |
| *Alistipes* | gene-*Rasef* | 0.89 | 0.02 | genus | gene |
| *Alistipes* | gene-*Fas* | -0.94 | 0.00 | genus | gene |
| *Alloprevotella* | gene-*C2cd4b* | 0.83 | 0.04 | genus | gene |
| *Alternaria* | gene-Rab19 | -0.89 | 0.02 | genus | gene |
| *Alternaria* | gene-*Fas* | -0.83 | 0.04 | genus | gene |
| *Bacteroides* | gene-*Rpl21* | -0.83 | 0.04 | genus | gene |
| *Bacteroides* | gene-Rab19 | 0.89 | 0.02 | genus | gene |
| *Clostridium* | gene-*Etv4* | 0.89 | 0.02 | genus | gene |
| *Clostridium* | gene-*Fas* | -0.83 | 0.04 | genus | gene |
| *Conocybe* | gene-*Zfand2a* | -0.94 | 0.00 | genus | gene |
| *Erysipelatoclostridium* | gene-*Rpl21* | 0.94 | 0.00 | genus | gene |
| *Lachnospiraceae_NK4A136_group* | gene-*Zfand2a* | 0.83 | 0.04 | genus | gene |
| *Lachnospiraceae_NK4A136_group* | gene-Rab19 | -0.83 | 0.04 | genus | gene |
| *Lachnospiraceae_UCG-006* | gene-*Tnfsf10* | -0.89 | 0.02 | genus | gene |
| *Lachnospiraceae_UCG-006* | gene-*Zfand2a* | 0.94 | 0.00 | genus | gene |
| *Lachnospiraceae_UCG-006* | gene-*Rasef* | 0.89 | 0.02 | genus | gene |
| *Lachnospiraceae_UCG-006* | gene-*Fas* | -0.94 | 0.00 | genus | gene |
| *Lactobacillus* | gene-*Etv4* | -1.00 | 0.00 | genus | gene |
| *Lactobacillus* | gene-*Tnfsf10* | 0.94 | 0.00 | genus | gene |
| *Lactobacillus* | gene-*Rasef* | -0.94 | 0.00 | genus | gene |
| *Lactobacillus* | gene-*Fas* | 0.89 | 0.02 | genus | gene |
| *Pichia* | gene-*C2cd4b* | -0.94 | 0.00 | genus | gene |
| *Pichia* | gene-*Rpl21* | -0.94 | 0.00 | genus | gene |
| *Pichia* | gene-*Tnfsf10* | 0.83 | 0.04 | genus | gene |
| *Pichia* | gene-*Rasef* | -0.83 | 0.04 | genus | gene |
| Prostaglandin C2 | *uncultured_bacterium_f_Muribaculaceae* | 0.89 | 0.02 | metabolite | genus |
| Prostaglandin C2 | *Erysipelatoclostridium* | -0.83 | 0.04 | metabolite | genus |
| Prostaglandin C2 | gene-*Rpl21* | -0.94 | 0.00 | metabolite | gene |
| Prostaglandin C2 | gene-*Tnfsf10* | 0.89 | 0.02 | metabolite | gene |
| Prostaglandin C2 | gene-*Rasef* | -0.89 | 0.02 | metabolite | gene |
| Prostaglandin C2 | Unclassified | 0.83 | 0.04 | metabolite | genus |
| Prostaglandin C2 | *Aspergillus* | -0.83 | 0.04 | metabolite | genus |
| Prostaglandin C2 | *Botryotrichum* | 0.89 | 0.02 | metabolite | genus |
| Prostaglandin C2 | *Pichia* | 0.89 | 0.02 | metabolite | genus |
| *Sampaiozyma* | gene-*Zfand2a* | 0.94 | 0.00 | genus | gene |
| *Unclassified* | gene-*Etv4* | -0.94 | 0.00 | genus | gene |
| *Unclassified* | gene-*Tnfsf10* | 0.89 | 0.02 | genus | gene |
| *Unclassified* | gene-*Rasef* | -0.89 | 0.02 | genus | gene |
| *uncultured_bacterium_f_Muribaculaceae* | gene-*Rpl21* | -0.94 | 0.00 | genus | gene |

Supplementary Table 10 The main compounds of SBE

| **No.** | ***t_R_* (min)** | **Formula** | **Adduct** | ***m/z*** | **Name** |
| --- | --- | --- | --- | --- | --- |
| 1 | 0.87 | C_6_H_12_O_7_ | [M-H]^-^ | 195.0507 | Gluconic acid* |
| 2 | 0.88 | C_5_H_9_NO_2_ | [M+H]^+^ | 116.0706 | Proline* |
| 3 | 0.92 | C_7_H_7_NO_2_ | [M+H]^+^ | 138.055 | Trigonelline* |
| 4 | 0.92 | C_7_H_12_O_6_ | [M-H]^-^ | 191.0561 | Quinic acid |
| 5 | 0.95 | C_7_H_13_NO_2_ | [M-H]^-^ | 142.0874 | Stachydrine |
| 6 | 0.97 | C_6_H_8_O_7_ | [M-H]^-^ | 191.0197 | Citric acid* |
| 7 | 1.01 | C_4_H_6_O_5_ | [M-H]^-^ | 133.0142 | Malic acid |
| 8 | 1.26 | C_5_H_4_O_3_ | [M-H]^-^ | 111.0088 | 2-furoic acid |
| 9 | 1.29 | C_5_H_6_O_4_ | [M-H]^-^ | 129.0193 | Mesaconic acid |
| 10 | 1.31 | C_5_ H_7_ N O_3_ | [M+H]^+^ | 130.0499 | Pyroglutamic Acid |
| 11 | 1.50 | C_4_ H_6_ O_4_ | [M-H]^-^ | 117.0193 | Methylmalonic acid |
| 12 | 1.51 | C_6_H_13_NO_2_ | [M+H]^+^ | 132.1019 | Isoleucine* |
| 13 | 2.46 | C_9_H_11_NO_2_ | [M+H]^+^ | 166.0863 | Phenylalanine* |
| 14 | 3.79 | C_16_H_18_O_9_ | [M-H]^-^ | 353.0878 | Neochlorogenic acid* |
| 15 | 3.79 | C_9_H_6_O_3_ | [M+H]^+^ | 163.039 | 7-hydroxycoumarine* |
| 16 | 3.91 | C_4_H_6_O_6_ | [M-H]^-^ | 149.0092 | Tartaric acid |
| 17 | 3.92 | C_9_H_8_O_4_ | [M-H]^-^ | 179.035 | Caffeic acid* |
| 18 | 4.58 | C_7_H_6_O_3_ | [M-H]^-^ | 137.0244 | Salicylic acid |
| 19 | 4.79 | C_16_H_18_O_9_ | [M-H]^-^ | 353.0878 | Chlorogenic acid* |
| 20 | 7.56 | C_21_H_18_O_12_ | [M-H]^-^ | 461.0725 | Scutellarin |
| 21 | 7.64 | C_15_H_10_O_6_ | [M+H]^+^ | 287.055 | Kaempferol* |
| 22 | 7.88 | C_21_H_18_O_11_ | [M-H]^-^ | 445.0776 | Baicalein 6-glucuronide |
| 23 | 8.12 | C_9_H_16_O_4_ | [M-H]^-^ | 187.0976 | Azelaic acid |
| 24 | 8.42 | C_10_H_10_O_2_ | [M+H]^+^ | 163.0754 | 4-methoxycinnamaldehyde |
| 25 | 9.74 | C_15_H_10_O_6_ | [M+H]^+^ | 285.0405 | Luteolin* |
| 26 | 10.77 | C_10_H_18_O | [M+H]^+^ | 155.143 | Isomenthone Dl |
| 27 | 20.64 | C_18_H_30_O_3_S | [M-H]^-^ | 325.1843 | 4-dodecylbenzenesulfonic acid |
| 28 | 25.95 | C_18_H_36_O_2_ | [M-H]^-^ | 283.2643 | Stearic acid |

Supplementary Table 11 Molecular docking results

| **Active ingredients** | **Targets** | **Affinity/(kJ/ mol)** |
| --- | --- | --- |
| Trigonelline | Fas | -4.8 |
| Mesaconic acid | Fas | -5.1 |
| Salicylic acid | Fas | -5.8 |
| Trigonelline | Tnsfs10 | -4.3 |
| Mesaconic acid | Tnsfs10 | -4.3 |
| Salicylic acid | Tnsfs10 | -4.8 |

**Supplementary Fig.2** Chemical structure of the active ingredient


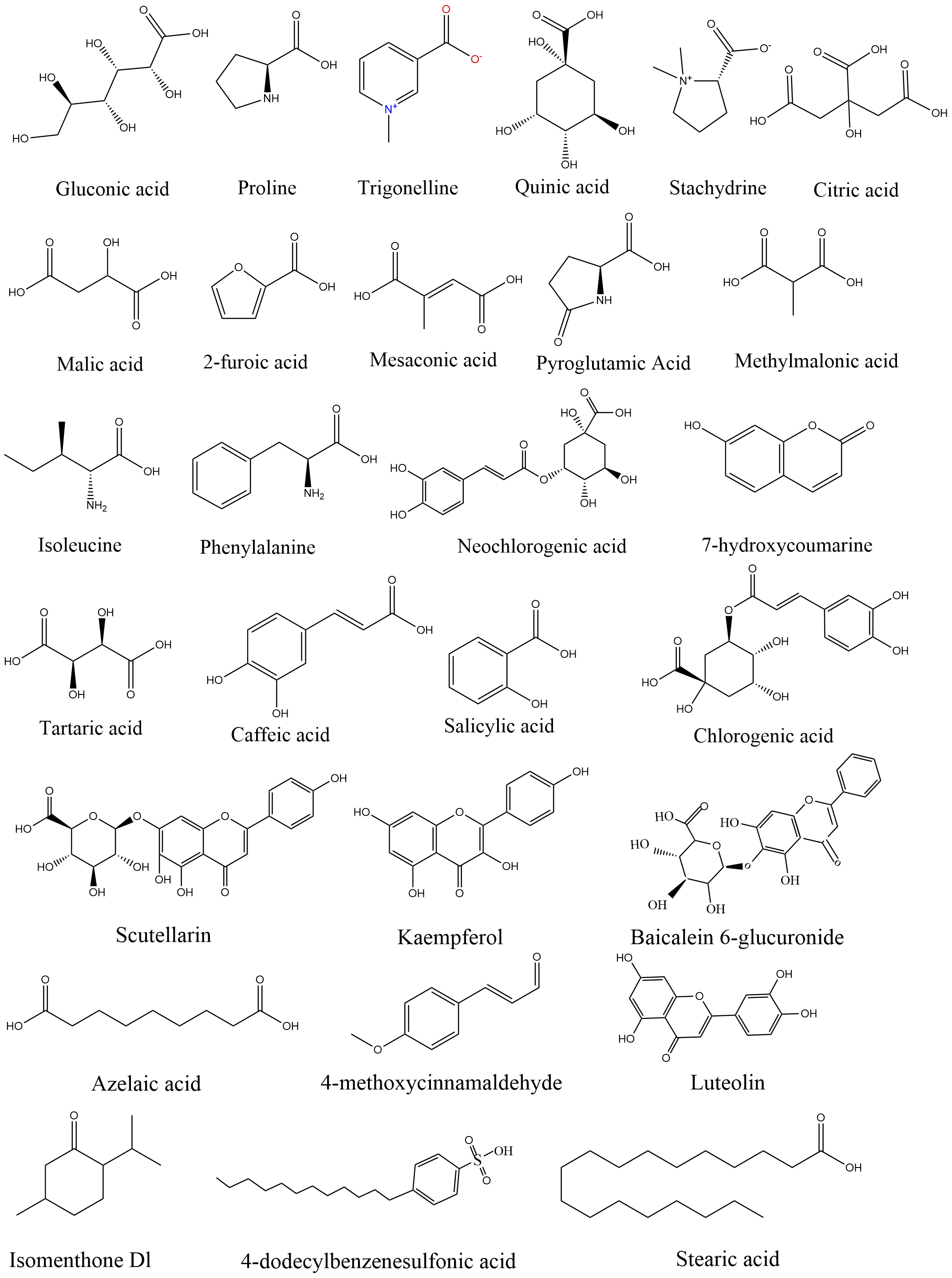


Supplementary Table 12 Abbreviation List of oxidative stress biomarker

| Abbreviation | Full Name |
| --- | --- |
| MDA | Malondialdehyde |
| PC | Protein carbonyl |
| SOD | Superoxide dismutase |
| GSH-Px | Glutathione peroxidase |
| CAT | Catalase |
| GSH | Glutathione |
